# The Arabidopsis circadian clock protein PRR5 interacts with and stimulates ABI5 to modulate abscisic acid signaling during seed germination

**DOI:** 10.1093/plcell/koab168

**Published:** 2021-06-21

**Authors:** Milian Yang, Xiao Han, Jiajia Yang, Yanjuan Jiang, Yanru Hu

**Affiliations:** 1 CAS Key Laboratory of Tropical Plant Resources and Sustainable Use, Xishuangbanna Tropical Botanical Garden, Chinese Academy of Sciences, Kunming, Yunnan 650223, China; 2 Center of Economic Botany, Core Botanical Gardens, Chinese Academy of Sciences, Kunming, Yunnan 650223, China; 3 University of Chinese Academy of Sciences, Beijing 100049, China

## Abstract

Seed germination and postgerminative growth require the precise coordination of multiple intrinsic and environmental signals. The phytohormone abscisic acid (ABA) suppresses these processes in *Arabidopsis thaliana* and the circadian clock contributes to the regulation of ABA signaling. However, the molecular mechanism underlying circadian clock-mediated ABA signaling remains largely unknown. Here, we found that the core circadian clock proteins PSEUDO-RESPONSE REGULATOR5 (PRR5) and PRR7 physically associate with ABSCISIC ACID-INSENSITIVE5 (ABI5), a crucial transcription factor of ABA signaling. PRR5 and PRR7 positively modulate ABA signaling redundantly during seed germination. Disrupting PRR5 and PRR7 simultaneously rendered germinating seeds hyposensitive to ABA, whereas the overexpression of *PRR5* enhanced ABA signaling to inhibit seed germination. Consistent with this, the expression of several ABA-responsive genes is upregulated by PRR proteins. Genetic analysis demonstrated that PRR5 promotes ABA signaling mainly dependently on ABI5. Further mechanistic investigation revealed that PRR5 stimulates the transcriptional function of ABI5 without affecting its stability. Collectively, our results indicate that these PRR proteins function synergistically with ABI5 to activate ABA responses during seed germination, thus providing a mechanistic understanding of how ABA signaling and the circadian clock are directly integrated through a transcriptional complex involving ABI5 and central circadian clock components.

## Teaser

PRR proteins physically interact with and stimulate ABI5 transcription factor to integrate circadian clock and ABA signaling during seed germination in Arabidopsis.

## Introduction

Seed germination and subsequent seedling establishment, two crucial developmental stages in flowering plants, require the precise coordination of multiple environmental and intrinsic signals. Among them, the phytohormone abscisic acid (ABA) is a pivotal signal that represses germination and subsequent seedling establishment, and stimulates seed maturation and dormancy in *Arabidopsis thaliana* (Finkelstein et al., 2002, 2008; Gubler et al., 2005; Finch-Savage and Leubner-Metzger, 2006). The presence of ABA is sensed by the PYRABACTIN RESISTANCE1 (PYR1)/PYR1-LIKE (PYL)/REGULATORY COMPONENT OF ABA RECEPTOR family of proteins (Ma et al., 2009; Miyazono et al., 2009; Nishimura et al., 2009; Park et al., 2009; Santiago et al., 2009). The recognition of ABA by these receptors leads to the repression of the co-receptor, type 2C protein phosphatases (PP2Cs), permitting the activation of a group of specific kinases termed SNF1-RELATED KINASE2 (SnRK2s; Ma et al., 2009; Park et al., 2009; Cutler et al., 2010; Zhao et al., 2020). SnRK2s subsequently phosphorylate and stabilize downstream regulators, such as the basic leucine zipper (bZIP)-type transcription factor ABSCISIC ACID-INSENSITIVE5 (ABI5) and its homologs ABSCISIC ACID-RESPONSIVE ELEMENT BINDING FACTORs, to mediate the expression of ABA-responsive genes (Kobayashi et al., 2005; Furihata et al., 2006; Fujii et al., 2007; Fujii and Zhu, 2009; NakashiMa et al., 2009).

The ABI5 transcription factor, mainly expressed in dry seeds and strongly induced by ABA, plays a critical role in ABA-inhibited seed germination and postgerminative growth (Finkelstein, 1994; Finkelstein and Lynch, 2000a; Lopez-Molina and Chua, 2000; Lopez-Molina et al., 2001, 2002; Brocard et al., 2002; Finkelstein et al., 2005; Skubacz et al., 2016; Fan et al., 2019). ABI5 is tightly regulated through protein posttranslational modifications (Stone et al., 2006; Garcia et al., 2008; Miura et al., 2009; Lee et al., 2010; Liu and Stone, 2010; Albertos et al., 2015; Yu et al., 2015; Lynch et al., 2017; Ji et al., 2019). For instance, ABI5 is activated through phosphorylation by the SnRK2s and other related kinases in response to ABA but repressed by PP6 (Kobayashi et al., 2005; Furihata et al., 2006; Fujii et al., 2007; Fujii and Zhu, 2009; NakashiMa et al., 2009; Dai et al., 2013; Hu and Yu, 2014; Zhou et al., 2015; Chen et al., 2021). ABI5 also acts as a key integrator between ABA and other signaling pathways during seed germination and postgerminative growth (Lim et al., 2013; Yu et al., 2015; Kim et al., 2016; Yang et al., 2016; Hu et al., 2019; Ju et al., 2019; Pan et al., 2020). For example, the kinase BRASSINOSTEROID-INSENSITIVE2 phosphorylates ABI5 to mediate the antagonism of brassinosteroids to ABA during seed germination (Hu and Yu, 2014), and cytokinin promotes degradation of ABI5 via the 26S proteasome pathway to antagonize ABA-inhibited cotyledon greening (Guan et al., 2014). Although much progress has been made in recent years, a comprehensive understanding of the transcriptional mechanisms underlying the crosstalk between ABA and other critical signals during seed germination remain elusive. 

The circadian clock is an endogenous time-keeping system that provides an adaptive advantage to higher plants by synchronizing internal biological processes with external daily environmental cycles (Dunlap, 1999; Dodd et al., 2005; Pruneda-Paz and Kay, 2010; Atkins and Dodd, 2014; Hsu and Harmer, 2014; Grundy et al., 2015; Sanchez and Kay, 2016; Webb et al., 2019; Simon et al., 2020). The oscillatory mechanism of the clock is based on transcriptional–translational feedback loops that connect morning- and evening-phase circuits (Harmer, 2009; Pokhilko et al., 2012; Carré and Veflingstad, 2013; Hsu and Harmer, 2014; Greenham and McClung, 2015; Uehara et al., 2019; Nakamichi, 2020). In the feedback loop, the genes encoding MYB transcription factors CIRCADIAN CLOCK-ASSOCIATED1 (CCA1) and LATE ELONGATED HYPOCOTYL (LHY) are expressed in the early morning (Wang and Tobin, 1998; Harmer, 2009), and CCA1 and LHY directly suppress the transcription of the pseudo-response regulator genes *PRR9*, *PRR7*, *PRR5*, and *TIMING OF CAB EXPRESSION1* (*TOC1*, also known as *PRR1*; Harmer et al., 2000; Matsushika et al., 2000; Strayer et al., 2000; Alabadi et al., 2001; Farré and Liu, 2013; Adams et al., 2015). These *PRR* genes are expressed when LHY and CCA1 protein levels decrease and, in turn, the PRR proteins act to inhibit *LHY* and *CCA1* transcription until the following morning (Alabadi et al., 2001; Perales and Más, 2007; Nakamichi et al., 2010, 2012; Gendron et al., 2012; Wang et al., 2010, 2013; Li et al., 2020a, 2020b).

The circadian clock integrates multiple internal and external signals to modulate plant growth, development, and physiology, such as photomorphogenesis, flowering, leaf senescence, and stress responses (Dunlap, 1999; Yamamoto et al., 2003; Dodd et al., 2005; Nakamichi et al., 2005, 2007, 2009; FukushiMa et al., 2009; Pruneda-Paz and Kay, 2010; Liu et al., 2013; Atkins and Dodd, 2014; Hsu and Harmer, 2014; Sanchez and Kay, 2016; Frank et al., 2018; Kim et al., 2020; Li et al., 2020a; Simon et al., 2020). Moreover, a close relationship between circadian clock and ABA biosynthesis or signaling has been reported in Arabidopsis*.* For instance, the circadian clock is involved in the production of ABA, thereby conferring a competitive advantage to the plant against drought, heat, salinity, and osmotic stresses (Burschka et al., 1983; Nováková et al., 2005; Lee et al., 2006; FukushiMa et al., 2009; Nakamichi et al., 2009; McAdam et al., 2011; Grundy et al., 2015; Adams et al., 2018). Several key genes that encode ABA biosynthetic enzymes, such as *NINE-CIS-EPOXYCAROTENOID DIOXYGENASE3* and *ABA DEFICIENT2*, exhibit circadian rhythmicity (Covington et al., 2008; FukushiMa et al., 2009; Penfield and Hall, 2009; Seung et al., 2012; Adams et al., 2018), and many ABA signaling components and downstream-responsive genes are rhythmically expressed (Covington et al., 2008; Michael et al., 2008; Mizuno and Yamashino, 2008; Penfield and Hall, 2009; Seung et al., 2012; Liu et al., 2013). LHY and CCA1 transcription factors have been shown to bind the promoter sequences of several genes critical for ABA biosynthesis and signaling (Adams et al., 2018). PRR5, PRR7, and PRR9 are also involved in ABA biosynthesis and signaling, and the content of ABA increases in *prr5 prr7 prr9* triple mutant seedlings (18-day-old plants; FukushiMa et al., 2009; Liu et al., 2013; Footitt et al., 2017). Moreover, multiple circadian clock proteins (e.g. CCA1, LHY, and TOC1) play important roles in seed dormancy and integrate environmental signaling controlling dormancy release in Arabidopsis (Penfield and Hall, 2009). Nevertheless, the exact molecular mechanisms underlying the circadian regulation of ABA responses during seed germination are still not fully understood.

In this study, we aimed to discover transcriptional regulation details of circadian clock-mediated ABA signaling during seed germination. We used the yeast two-hybrid system to identify potential ABI5-interacting partners involved in the circadian clock, and found that PRR5 and PRR7 physically associate with ABI5 in yeast (*Saccharomyces cerevisiae*) and in planta. Phenotypic analysis showed that PRR5, PRR7 as well as PRR9 positively regulate ABA signaling redundantly during seed germination. The *prr5 prr7* double mutant and *prr5 prr7 prr9* triple mutant are hyposensitive to ABA during seed germination. Conversely, overexpressing *PRR5* causes germinating seeds to become ABA-hypersensitive. Further genetic analysis demonstrated that the ABA hypersensitivity of *PRR5*-overexpressing plants requires functional ABI5 protein. Consistently, the mechanistic investigations revealed that PRR5 stimulates the transcriptional function of ABI5 to modulate downstream target genes. Together, our findings indicate that these PRR proteins act synergistically with ABI5 to positively regulate the ABA responses during seed germination and provide a mechanistic understanding of the crosstalk between the circadian clock and ABA signaling.

## Results

### ABI5 physically interacts with PRR5 and PRR7

**Figure 1:**
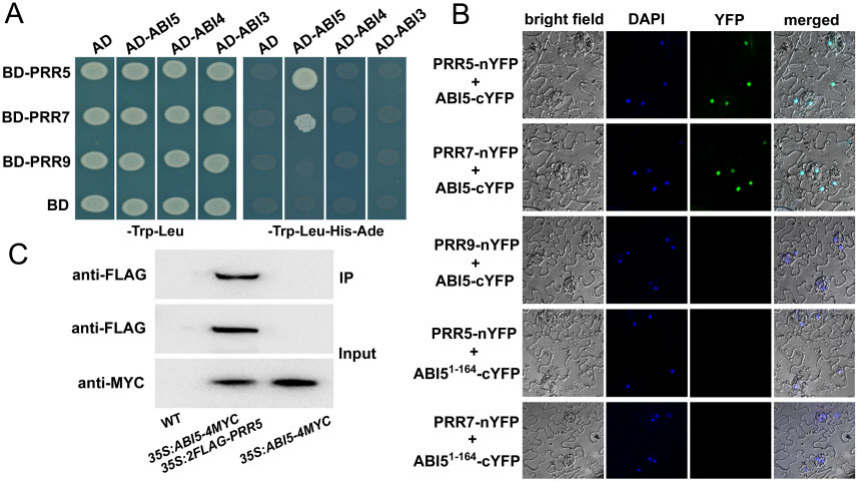
Physical interactions of ABI5 with PRR5 and PRR7. A, Yeast two-hybrid screening assays. Interaction of ABI5 with PRR5 or PRR7 is indicated by the ability of yeast cells to grow on dropout medium lacking Leu, Trp, His, and Ade for 4 d after plating. pGBKT7 (BD) and pGADT7 (AD) were used as negative controls. B, BiFC assays. Fluorescence was observed in the nuclear compartment of transformed *N. benthamiana* cells, resulting from the complementation of ABI5-cYFP with PRR5-nYFP or PRR7-nYFP. No signal was obtained for the negative controls in which ABI5-cYFP was coexpressed with PRR9-nYFP, and ABI5^1-164^-cYFP (the sequence encoding the N-terminal domain of ABI5 fused with cYFP) was coexpressed with PRR5-nYFP or PRR7-nYFP. Nuclei are indicated by DAPI staining. C, CoIP assays. MYC-fused ABI5 was immunoprecipitated using an anti-MYC antibody (1:250) and the coimmunoprecipitated protein was detected using an anti-FLAG antibody (1:10,000). Protein input for MYC-ABI5 in immunoprecipitated complexes was also detected and is shown. Experiments were repeated three times with similar results.

The ABI5 transcription factor is a critical modulator of ABA signaling, which represses seed germination and early seedling growth. Importantly, ABI5 also may function as a crucial interaction node to integrate ABA signaling and other pathways. To further investigate the molecular mechanisms underlying the circadian regulation of ABA signaling during seed germination, we performed yeast two-hybrid analysis to identify possible physical interactions between ABI5 and core components of the circadian clock, including CCA1, LHY, PRR9, PRR7, PRR5, PRR3, and TOC1. The full-length ABI5 was fused to the Gal4 activation domain (AD) of the prey vector (AD-ABI5) and the full-length of the clock proteins were ligated with the Gal4 DNA-binding domain (BD) of the bait vector (BD-CCA1, BD-LHY, BD-PRR, and BD-TOC1). As shown in Figure 1A, ABI5 physically associated with PRR5 and PRR7 in the yeast two-hybrid system, and no interaction was detected between ABI5 and CCA1, LHY, PRR9, PRR3, or TOC1 (Figure 1A; Supplemental Figure S1). Parallel experiments showed that ABI3 and ABI4, two other key transcription factors involved in ABA signaling (Giraudat et al., 1992; Finkelstein, 1994; Finkelstein et al., 1998; Söderman et al., 2000), did not interact with PRR5 and PRR7 in yeast (Figure 1A), supporting the specificity of the interactions of ABI5 with PRR5 and PRR7.

To corroborate that ABI5 interacts with PRR5 and PRR7 in plant cells, we used the bimolecular fluorescence complementation (BiFC) assay. The full-length coding sequence (CDS) of ABI5 was ligated with the sequence encoding the C-terminal yellow fluorescent protein (YFP) fragment driven by the Cauliflower mosaic virus (CaMV) 35S promoter to generate ABI5-cYFP, whereas the full-length PRR5, PRR7, and PRR9 were fused with the sequence encoding the N-terminal YFP fragment to produce PRR5-nYFP, PRR7-nYFP, and PRR9-nYFP. When ABI5-cYFP was coexpressed transiently with PRR5-nYFP or PRR7-nYFP in leaf cells of wild tobacco (*Nicotiana benthamiana*), strong YFP fluorescence was detected in the nucleus of the transformed cells, as revealed by staining with 4′,6-diamidino-2-phenylindole (DAPI; Figure 1B). No YFP signal was observed in the negative control assays in which ABI5-cYFP was coexpressed with PRR9-nYFP and ABI5^1–164^-cYFP (the sequence encoding the N-terminal amino acid residues 1–164 of ABI5 fused to *cYFP*) was coexpressed with PRR5-nYFP or PRR7-nYFP (Figure 1B). Moreover, as shown in Figure 1C, a coimmunoprecipitation (CoIP) assay provided further evidence of the association between ABI5 and PRR5 in transgenic Arabidopsis simultaneously overexpressing *ABI5* and *PRR5* (*35S:ABI5-4MYC/35S:2FLAG-PRR5*), which was constructed by introducing a *PRR5* overexpression construct (*35S:2FLAG-PRR5*) into previously described *35S:ABI5-4MYC* plants (containing a functional ABI5-4MYC construct driven by the CaMV 35S promoter; Chen et al., 2012; Hu et al., 2019). Collectively, these results demonstrate that ABI5 physically interacts with PRR5 and PRR7, implying that PRR5 and PRR7 may function as two interacting partners of ABI5 to mediate ABA responses during seed germination.

### The bZIP domain of ABI5 and the C-terminal fragment of PRR5 are responsible for the interaction

**Figure 2:**
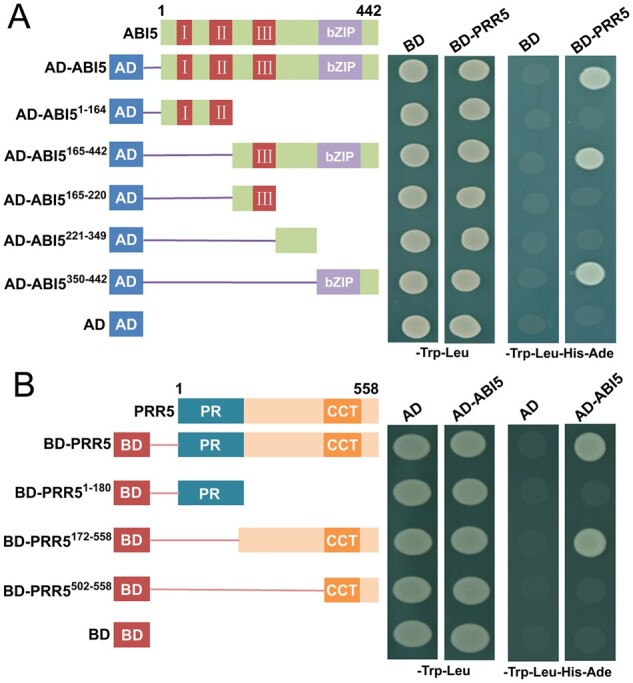
Yeast two-hybrid screening assays to identify ABI5 and PRR5 regions required for their interaction. A, The bZIP domain of ABI5 interacts with PRR5. Left: Diagram of full-length and truncated ABI5 constructs with specific deletions. Right: Interaction is indicated by the ability of cells to grow on dropout medium lacking Leu, Trp, His, and Ade for 4 days after plating. pGBKT7 (BD) and pGADT7 (AD) were used as negative controls. B, The C-terminal fragment of PRR5 interacts with ABI5. Left: Diagram of full-length and truncated PRR5 constructs with specific deletions. Right: Interaction is indicated by the ability of cells to grow on dropout medium lacking Leu, Trp, His, and Ade for 4 days after plating. BD and AD were used as negative controls. BD and AD vectors were used as negative controls.

To identify the region of ABI5 essential for the interaction with PRR5, we fused five truncated ABI5 variants to the Gal4 AD of the prey vector (Figure 2A) and examined the interaction between these variants and PRR5 by yeast two-hybrid analysis. As shown in Figure 2A, deletion of the N-terminal amino acid residues 1–164 of ABI5 (AD-ABI5^165–442^) did not affect the ABI5–PRR5 interaction, whereas deletion of the 278 C-terminal residues of ABI5 that harbor the bZIP domain (AD-ABI5^1–164^) completely abolished the ABI5–PRR5 interaction (Figure 2A). This result shows that the C-terminal region of ABI5 was required for its interaction with PRR5. Further mapping revealed that the 93 amino acids spanning the C-terminal bZIP domain were specifically involved in the ABI5–PRR5 interaction, because an ABI5 variant in which the N-terminal amino acids 1–349 were deleted (AD-ABI5^350–442^) could still physically associate with PRR5 (Figure 2A).

Similarly, to determine the PRR5 region critical for the interaction with ABI5, we truncated the sequences of PRR5 to obtain variants with the N-terminal PR domain, the C-terminal fragment, or the CCT domain (Figure 2B; Kiba et al., 2007). We fused the truncated PRR5 sequences to the Gal4 DNA-BD of the pGBKT7 vector as baits and performed directed yeast two-hybrid analysis. As shown in Figure 2B, the N-terminal PR domain (BD-PRR5^1–180^) and the CCT domain (BD-PRR5^502–558^) did not interact with ABI5, whereas the C-terminal fragment (BD-PRR5^172–558^) strongly interacted with ABI5. These results demonstrate that the entire C-terminal region of PRR5 is crucial in forming the ABI5–PRR5 interaction.

### The *prr5 prr7* double and *prr5 prr7 prr9* triple mutants are hyposensitive to ABA during seed germination

**Figure 3:**
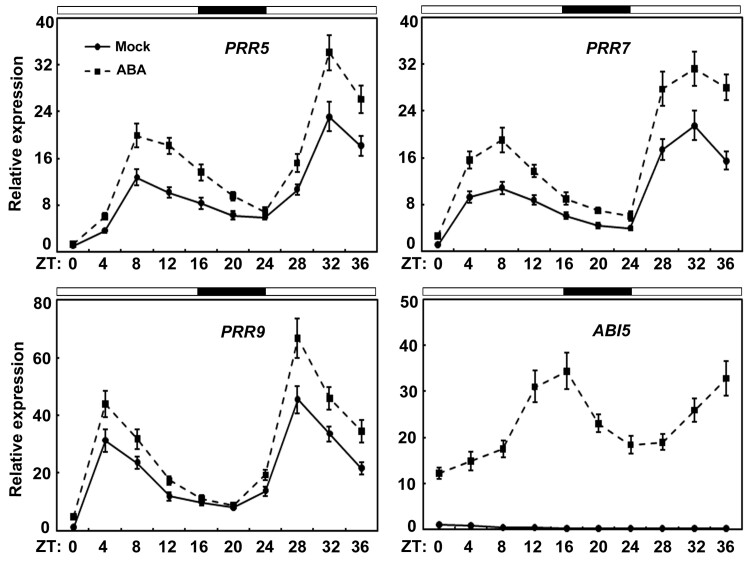
Expression of *PRR5, PRR7, PRR9*, and *ABI5* in response to ABA during seed germination. RT-qPCR analysis of the ABA-induced expression of *PRR5, PRR7, PRR9*, and *ABI5* in germinating wild-type (WT) seeds. Total RNA was extracted from three different batches of germinating seeds (2 days, harvested from ZT0 to ZT36) of WT with or without (Mock) 0.5-μM ABA treatment grown under 16-h-light/8-h-dark for indicated times. Time is expressed as hours from dawn (ZT0). The *PP2A* (AT1G13320) gene was used as control. Error bars show sd from three independent biological replicates. Values are means ± sd.

Previous studies showed that PRR proteins are core clock components in Arabidopsis that regulate multiple physiological processes, such as photomorphogenesis, flowering, and stress responses (Yamamoto et al., 2003; Nakamichi et al., 2005, 2007, 2009; Farré and Liu, 2013; Liu et al., 2013; Li et al., 2020a; Yuan et al., 2021)*.* Because PRR5 and PRR7 physically interact with the ABI5 transcription factor, we queried whether they are involved in ABI5-mediated ABA signaling during seed germination. To test this possibility, we first analyzed the expression of *PRR5*, *PRR7*, as well as *PRR9* in ABA-treated wild-type seeds. As shown in Figure 3, the expression of *PRR5, PRR7*, and *PRR9* was rhythmic and responsive to ABA during the early stage of germination (Figure 3). Similarly, we detected the expression of *ABI5* in wild-type germinating seeds, and found that the transcript levels of *ABI5* also displayed a diel pattern in response to ABA (Figure 3). Then, we evaluated the germination of the loss-of-function *prr5* (*prr5-1* and *prr5-2*) and *prr7* (*prr7-1* and *prr7-2*) single mutants on half-strength Murashige and Skoog (MS) supplemented with different concentrations of ABA. As shown in Supplemental Figure S2, A and B, seeds of *prr5* and *prr7* single mutants displayed germination and greening percentages in response to ABA which were similar to those of wild-type seeds. To avoid the effects of sucrose and/or nitrate on seed germination (Garciarrubio et al., 1997; Finkelstein and Lynch, 2000b; Dekkers et al., 2004; Alboresi et al., 2005; Dave et al., 2011), we also analyzed the phenotypes of *prr5* and *prr7* single mutants on water agar medium and found that these mutants behaved like the wild-type upon ABA treatment during seed germination (Supplemental Figure S2, C and D). This finding shows that disruption of PRR5 or PRR7 alone had little effect on ABA responses during seed germination and subsequent seedling establishment.

**Figure 4:**
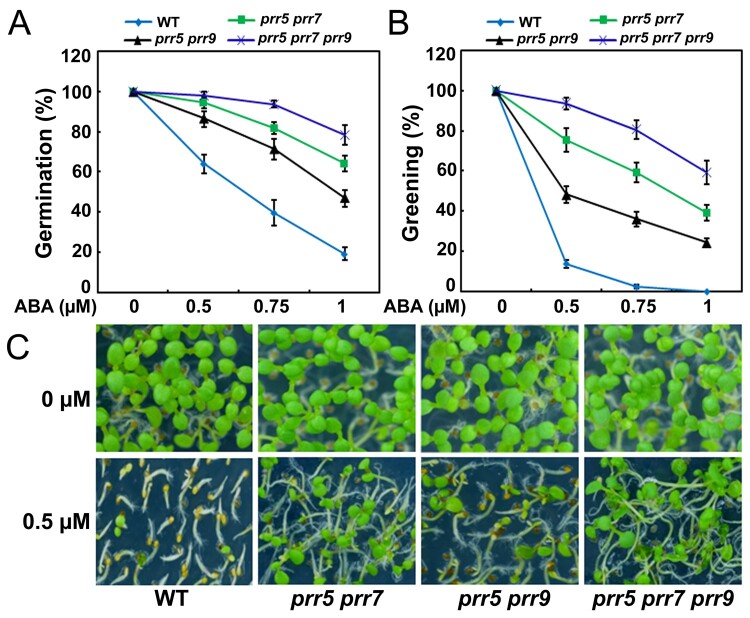
ABA responses of *prr5 prr7*, *prr5 prr9*, and *prr5 prr7 prr9* mutants during seed germination. A, Germination of the WT, *prr5 prr7*, *prr5 prr9*, and *prr5 prr7 prr9* mutants. Seed germination was recorded 2 days after stratification on half-strength MS medium supplemented with different concentrations of ABA. B, Cotyledon greening of the WT, *prr5 prr7*, *prr5 prr9*, and *prr5 prr7 prr9* mutants. Cotyledon greening was scored 4.5 days after stratification on half-strength MS medium supplemented with different concentrations of ABA. Experiments were performed seven times by analyzing different batches of seeds. Each batch of seeds of WT, *prr5 prr7*, *prr5 prr9*, and *prr5 prr7 prr9* mutants was pooled from more than 60 independent plants. For each biological replicate, more than 120 seeds were examined. Values are means ± sd. C, Seedlings of WT, *prr5 prr7*, *prr5 prr9*, and *prr5 prr7 prr9* mutants 4.5 days after germination on half-strength MS medium containing 0.5-μM ABA.

Because PRR5 and PRR7 play partially overlapping roles in the circadian clock, we hypothesized that they may mediate ABA signaling redundantly during seed germination. To test this speculation, we genetically crossed *prr5-1* with *prr7-2* to generate a *prr5 prr7* double mutant and evaluated its performance in half-strength MS medium containing different concentrations of ABA. As shown in Figure 4, A–C, the progeny of the *prr5 prr7* double mutant were hyposensitive to ABA during seed germination and showed much higher germination and greening than the wild-type. PRR9 is a close homolog of PRR5 and PRR7 in regulating the circadian clock and various other physiological processes (Nakamichi et al., 2005, 2007, 2009; Farré and Liu, 2013). To test whether PRR9 acts together with PRR5 and PRR7 in ABA signaling, we examined the phenotypes of the *prr5 prr9* double mutant and *prr5 prr7 prr9* triple mutant. The results showed that the *prr5 prr7 prr9* triple mutant had much higher germination and greening percentages than the wild-type and the *prr5 prr7* and *prr5 prr9* double mutants (Figure 4, A–C). We also analyzed the phenotypes of these mutants on water agar medium supplemented with ABA and found that the seeds of the *prr5 prr7*, *prr5 prr9*, and *prr5 prr7 prr9* mutants also were more hyposensitive to ABA than the seeds of the wild-type during seed germination (Supplemental Figure S3).

**Figure 5:**
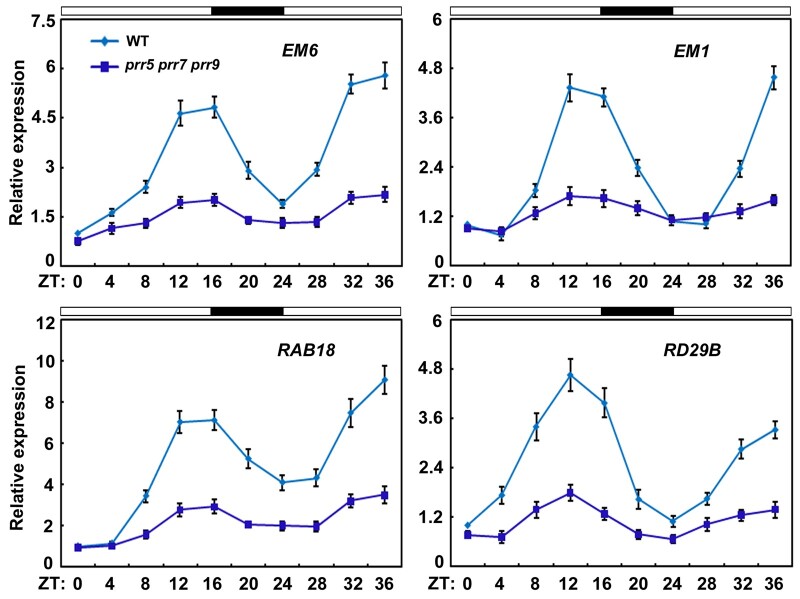
Expression levels of several ABA-responsive Genesin *prr5 prr7 prr9*. RT-qPCR analysis of the ABA-induced expression of *EM6, EM1, RAB18*, and *RD29B* in the WT and *prr5 prr7 prr9.* Total RNA was extracted from three different batches of germinating seeds (2 days, harvested from ZT0 to ZT36) of WT and *prr5 prr7 prr9* with 0.5-μM ABA treatment grown under 16-h-light/8-h-dark for indicated times. Time is expressed as hours from dawn (ZT0). The *PP2A* (AT1G13320) gene was used as control. Error bars show sd from three independent biological replicates. Values are means ± sd.

To confirm the *prr5 prr7 prr9* triple mutant phenotype in response to ABA, we examined the expression of several well-characterized ABA-responsive genes in ABA-treated germinating seeds of the *prr5 prr7 prr9* triple mutant, including *LATE EMBRYOGENESIS ABUNDANT 6* (*EM6*)*, EM1*, *RESPONSIVE TO ABA 18* (*RAB18*), and *RESPONSIVE TO DESICCATION 29B* (*RD29B*). In wild-type germinating seeds, *EM6*, *EM1*, *RAB18*, and *RD29B* displayed circadian expression patterns, implying that these genes may be modulated by the circadian clock (Figure 5). However, in the *prr5 prr7 prr9* triple mutant, the *EM1*, *EM6*, *RAB18*, and *RD29B* transcript levels decreased compared with those in the wild-type, and the circadian amplitude of their expression was greatly attenuated (Figure 5). These results suggest that PRR5, PRR7, and PRR9 may upregulate the expression of these ABA-induced genes during seed germination. Taken together, these results show that PRR5, PRR7, and PRR9 may positively modulate ABA responses during seed germination.

### Overexpression of *PRR5* confers germinating seeds being ABA-hypersensitive

**Figure 6:**
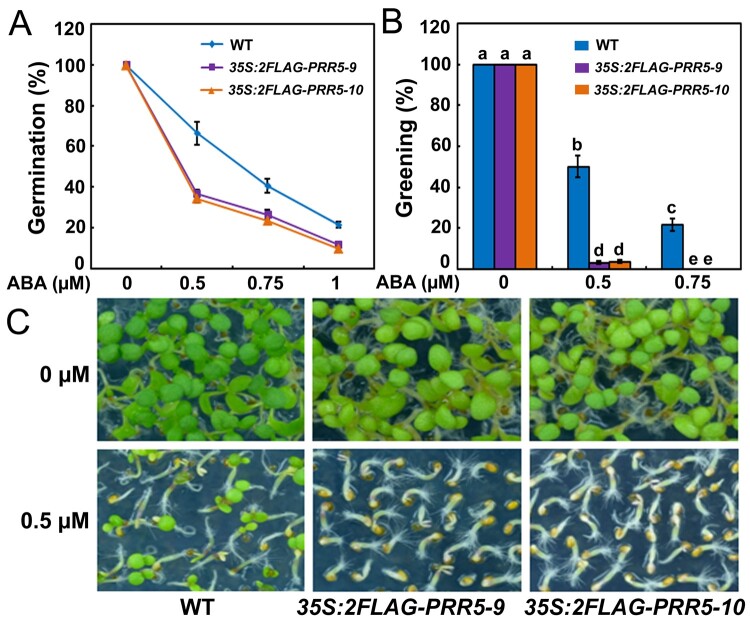
ABA responses of *PRR5*-overexpressing plants during seed germination. A, Germination of *PRR5*-overexpressing plants *35S:2FLAG-PRR5-9* and *35S:2FLAG-PRR5-10.* Seed germination was recorded 2 days after stratification on half-strength MS medium supplemented with different concentrations of ABA. B, Cotyledon greening of WT, *35S:2FLAG-PRR5-9*, and *35S:2FLAG-PRR5-10*. Cotyledon greening was scored 5 days after stratification on half-strength MS medium supplemented with 0.5- or 0.75-μM ABA. Experiments were performed five times by analyzing different batches of seeds. Each batch of seeds of WT, *35S:2FLAG-PRR5-9*, and *35S:2FLAG-PRR5-10* was pooled from more than 60 independent plants. For each biological replicate, more than 120 seeds were examined. Values are means ± sd. Bars with different letters are significantly different from each other (*P* < 0.05). Data were analyzed by analysis of variance (ANOVA). C, Seedlings of WT, *35S:2FLAG-PRR5-9*, and *35S:2FLAG-PRR5-10* 5 days after germination on half-strength MS medium containing 0.5-μM ABA.

To further analyze the role of PRR5 in ABA signaling during seed germination and postgerminative growth, we generated transgenic plants overexpressing *PRR5* (*35S:2FLAG-PRR5*) under the control of the CaMV 35S promoter. Reverse transcription-quantitative polymerase chain reaction (RT-qPCR) analysis showed that some of overexpressing lines had elevated levels of *PRR5* transcripts under normal growth condition (Supplemental Figure S4). We selected the homozygous *35S:2FLAG-PRR5-9* and *35S:2FLAG-PRR5-10* transgenic plants for further analysis (Supplemental Figure S4). Consistent with previous studies (Sato et al., 2002; Murakami et al., 2004), the F4 progeny of these transgenic plants exhibited an early flowering phenotype compared with the wild-type. We investigated the performances of *35S:2FLAG-PRR5-9* and *35S:2FLAG-PRR5-10* on half-strength MS medium with various concentrations of ABA during seed germination. As shown in Figure 6A, the progeny of *35S:2FLAG-PRR5-9* and *35S:2FLAG-PRR5-10* had much lower germination percentages than the wild-type at the ABA concentration tested. Moreover, the seeds of *35S:2FLAG-PRR5-9* and *35S:2FLAG-PRR5-10* showed significantly less greening than the seeds of the wild-type (Figure 6, B and C). Likewise, on water agar media containing ABA, *35S:2FLAG-PRR5-9* and *35S:2FLAG-PRR5-10* were also more sensitive to ABA than the wild-type during seed germination (Supplemental Figure S5). Thus, the overexpression of *PRR5* enhances ABA responses during seed germination, which further supports the notion that PRR5 positively mediates ABA signaling to repress seed germination and early seedling growth in Arabidopsis.

### Genetic interaction between *ABI5* and *PRR5*

**Figure 7:**
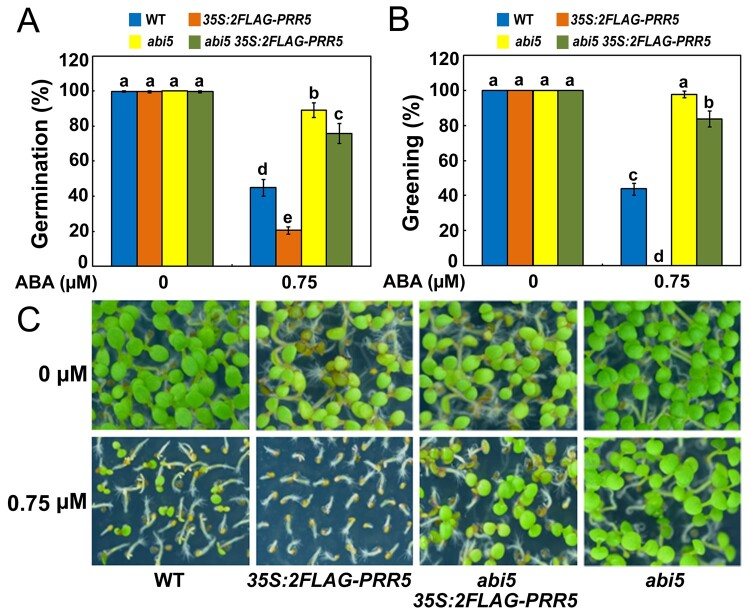
ABA hypersensitivity of *PRR5*-overexpressing plants requires functional ABI5. A, Germination of *PRR5*-overexpressing wild-type (*35S:2FLAG-PRR5-10*) and *abi5* (*abi5 35S:2FLAG-PRR5*) seeds. Seed germination was recorded 2 days after stratification on half-strength MS medium supplemented with 0.75-μM ABA. B, Cotyledon greening of WT, *35S:2FLAG-PRR5-10, abi5 35S:2FLAG-PRR5*, and *abi5*. Cotyledon greening was scored 6 days after stratification on half-strength MS medium supplemented with 0.75-μM ABA. Experiments were performed five times by analyzing different batches of seeds. Each batch of seeds of WT, *35S:2FLAG-PRR5-10*, *abi5 35S:2FLAG-PRR5*, and *abi5* was pooled from more than 60 independent plants. For each biological replicate, more than 120 seeds were examined. Values are means ± sd. Bars with different letters are significantly different from each other (*P* < 0.05). Data were analyzed by analysis of variance (ANOVA). C, Seedlings of WT, *35S:2FLAG-PRR5-10, abi5 35S:2FLAG-PRR5*, and *abi5* 7 days after germination on half-strength MS medium containing 0.75-μM ABA.

**Figure 8:**
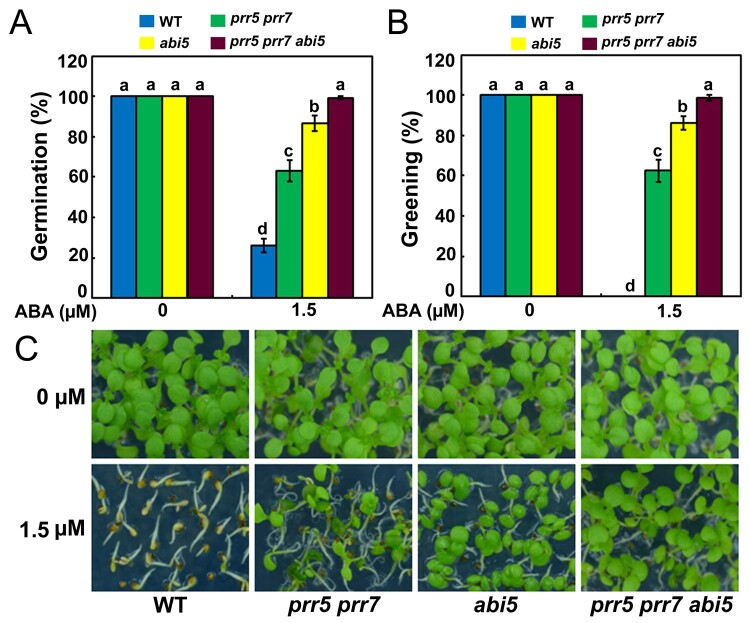
ABA responses of *prr5 prr7*, *abi5*, and *prr5 prr7 abi5* mutants during seed germination. A, Germination of the WT, *prr5 prr7*, *abi5*, and *prr5 prr7 abi5* mutants. Seed germination was recorded 3 days after stratification on half-strength MS medium supplemented with 1.5-μM ABA. B, Cotyledon greening of the WT, *prr5 prr7*, *abi5*, and *prr5 prr7 abi5* mutants. Cotyledon greening was scored 6 days after stratification on half-strength MS medium supplemented with 1.5-μM ABA. Experiments were performed five times by analyzing different batches of seeds. Each batch of seeds of the WT, *prr5 prr7*, *abi5*, and *prr5 prr7 abi5* mutants was pooled from more than 60 independent plants. For each biological replicate, more than 120 seeds were examined. Values are means ± sd. Bars with different letters are significantly different from each other (*P* < 0.05). Data were analyzed by analysis of variance (ANOVA). C, Seedlings of the WT, *prr5 prr7*, *abi5*, and *prr5 prr7 abi5* 6 days after germination on half-strength MS medium containing 1.5-μM ABA.

Having ascertained that PRR5 interacts with ABI5 and positively modulates ABA responses, we asked whether the action of PRR5 in mediating ABA signaling required functional ABI5. To test this possibility, we generated *abi5 35S:2FLAG-PRR5* plants by genetically crossing *35S:2FLAG-PRR5-10* with *abi5* (*abi5-1*), which is a loss-of-function mutant of *ABI5* in the Wassilewskija background (Finkelstein, 1994; Finkelstein and Lynch, 2000a) and was introduced into the Columbia (Col) background through backcrossing it with the Col wild-type six times (Hu et al., 2019). Similar to *abi5* seeds, progeny of *abi5 35S:2FLAG-PRR5* was also hyposensitive to ABA during seed germination, with much higher percentages of germination and greening compared with those of the wild-type and *35S:2FLAG-PRR5-10* plants (Figure 7, A–C). These results show that the ABA hypersensitivity of *35S:2FLAG-PRR5-10* requires a functional ABI5 transcription factor. However, the responses of *abi5 35S:2FLAG-PRR5* after exposure to ABA were different from those of the *abi5* mutant (Figure 7, A–C). To further elucidate the genetic relationship between *ABI5* and *PRR5* in ABA signaling, we crossed *abi5* with the *prr5 prr7* double mutant to produce a *prr5 prr7 abi5* triple mutant, and investigated its phenotype in the presence of ABA during seed germination. As shown in Figure 8, A–C, the *prr5 prr7 abi5* triple mutant had higher germination and greening percentages than *prr5 prr7* and *abi5* on half-strength MS medium containing 1.5-µM ABA, implying that PRR5 and PRR7 may associate with other proteins besides ABI5 to mediate ABA signaling.

### PRR5 stimulates the transcriptional function of ABI5

**Figure 9:**
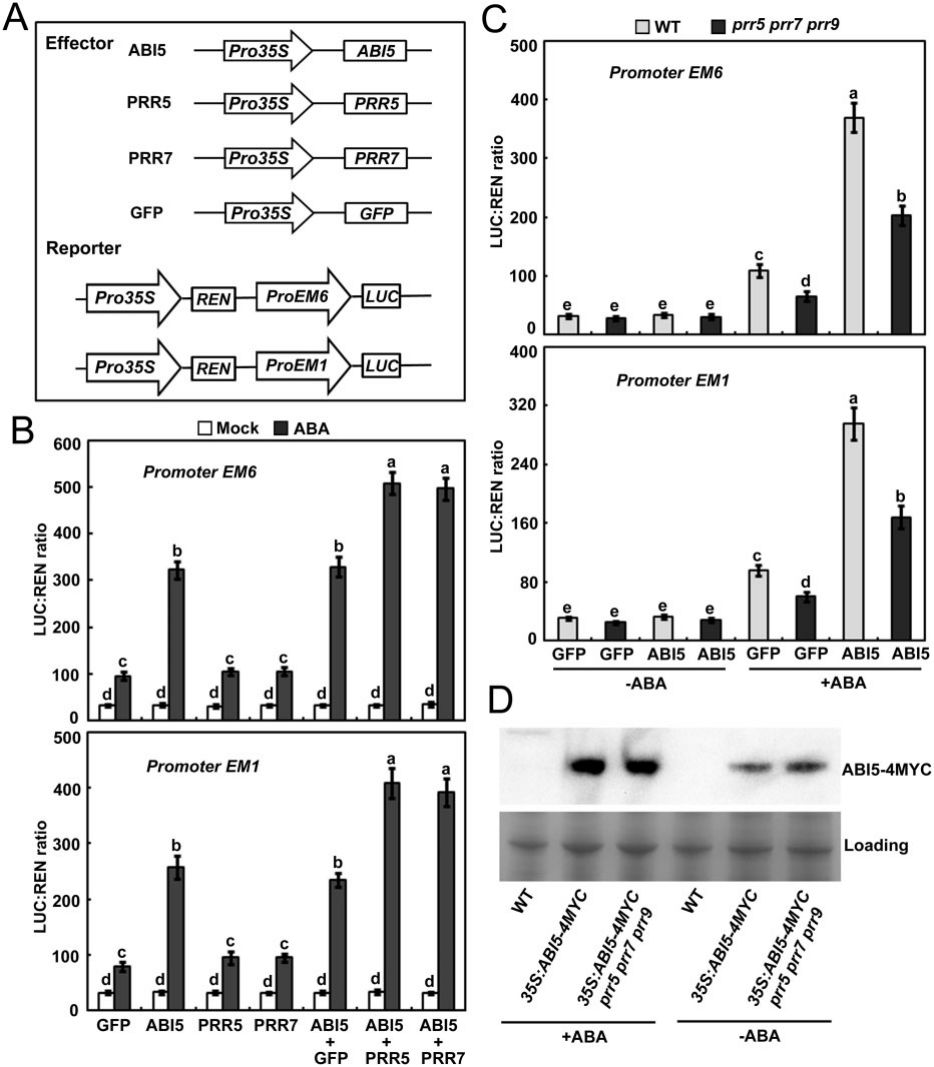
PRR5 promotes the transcriptional function of ABI5. A, Schematic of the effectors and reporters used in the transient transactivation assays. B, Transient dual-LUC reporter assays showing that PRR5 and PRR7 stimulate ABI5 to modulate the expression of *EM6* or *EM1* in response to 5-μM ABA. Error bars show sd from three biological replicates using different batches of wild-type plants; each replication was from different wild-type leaves of more than 50 plants. C, Transient transcriptional activity assays showing that activation of the *EM6* and *EM1* promoter by ABI5 is decreased in the *prr5 prr7 prr9* mutant in response to 5-μM ABA. Error bars indicate sd from three biological replicates using different batches of *prr5 prr7 prr9* mutants; each replication was from different leaves of more than 50 plants. Bars with different letters are significantly different from each other (*P* < 0.05). Data were analyzed by analysis of variance (ANOVA). D, Immunoblot analyzing the ABA-induced accumulation of ABI5 protein in the WT and *prr5 prr7 prr9* plants. Whole seedlings of 5-day-old WT, *35S:ABI5-4MYC*, and *35S:ABI5-4MYC prr5 prr7 prr9* were treated with 100-μM ABA for 6 h before protein extraction. The accumulation of ABI5-4MYC fused protein was detected by immunoblotting with an anti-MYC antibody. Experiments were repeated three times with similar results.

Recent studies have revealed that several interacting partners of ABI5 exert their regulatory effects mainly by stimulating or repressing the transcriptional function of ABI5 (Lim et al., 2013; Kim et al., 2016; Hu et al., 2019; Ji et al., 2019; Ju et al., 2019; Zhang et al., 2019; Zhao et al., 2019; Pan et al., 2020). Because PRR5 physically and genetically interacts with ABI5, we examined whether it also affects the ability of ABI5 to activate downstream targets. To test this, we initially investigated the possible regulatory effect of PRR5 on the transcriptional function of ABI5 in wild-type Arabidopsis mesophyll protoplasts using a dual-luciferase (LUC) reporter approach (Yoo et al., 2007). The effectors contained an *ABI5*, *PRR5*, *PRR7*, or *GFP* (green fluorescent protein) gene under the control of the CaMV 35S promoter (Figure 9A). Because *EM6* and *EM1* are direct downstream targets of ABI5 (Finkelstein and Lynch, 2000a; Lopez-Molina and Chua, 2000; Nakamura et al., 2001; Carles et al., 2002; Reeves et al., 2011), we fused their promoters with the *LUC* gene to produce reporter constructs (Figure 9A). Consistent with previous studies (Zhou et al., 2015; Pan et al., 2018; Hu et al., 2019), expression of ABI5 significantly increased the expression level of *LUC* driven by the *EM6* or *EM1* promoters in the presence of 5-µM ABA compared with the expression of GFP alone (Figure 9B). More importantly, the coexpression of PRR5 with ABI5 further enhanced the *LUC* expression level when compared with the coexpression of GFP and ABI5 (Figure 9B). Similar results were found when PRR7 was coexpressed with ABI5 in these assays (Figure 9B). These results suggest that PRR5 and PRR7 may stimulate the transcriptional function of ABI5 to modulate downstream *EM6* or *EM1* under ABA treatment.

**Figure 10:**
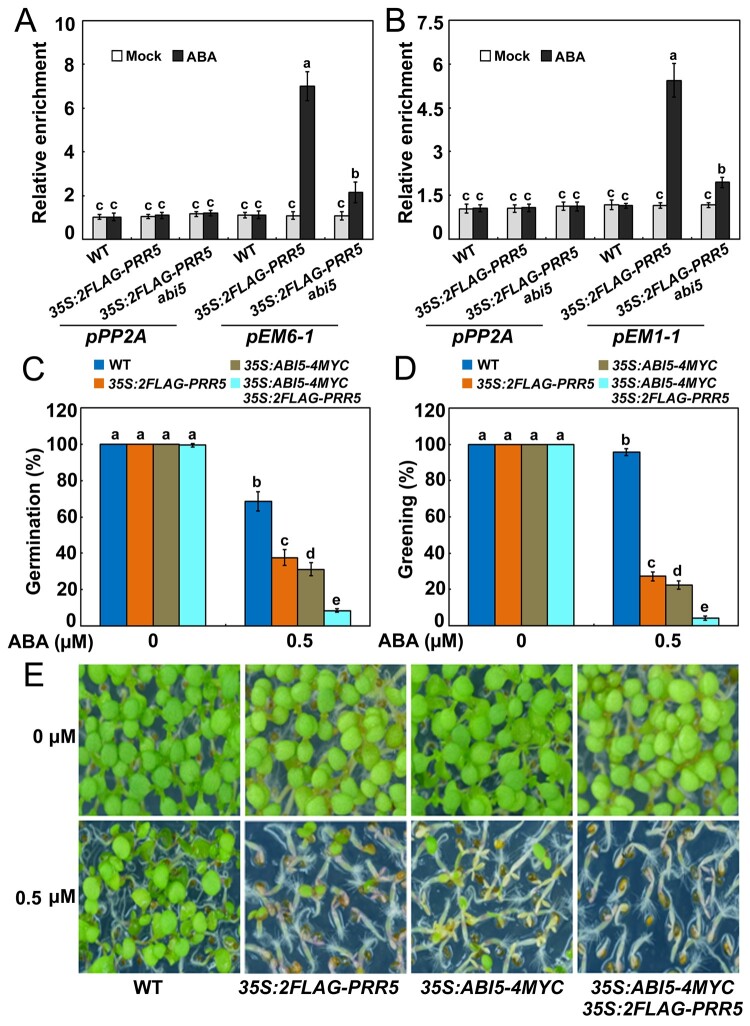
ABA hypersensitivity of *ABI5*-overexpressing plants is enhanced by *PRR5* overexpression during seed germination. A, B, ChIP-qPCR analysis of the relative enrichment of PRR5 on the promoter regions of *EM6* (*pEM6-1*) and *EM1* (*pEM1-1*). Three different batches of 0.5-μM ABA-treated (for 2.5 days) germinating seeds of *PRR5*-overexpressing WT (*35S:2FLAG-PRR5-10*) and *abi5* (*abi5 35S:2FLAG-PRR5*) pooled from more than 60 independent plants were used in ChIP using anti-FLAG antibody. qPCR data from the ChIP assay with anti-FLAG antibody with the *PP2A* (AT1G13320) promoter (*pPP2A*) as a negative control. Error bars show sd from three biological replicates using different batches of seeds, and different letters above the columns indicate significant differences based on analysis of variance (ANOVA; *P* < 0.05). C, Germination of *ABI5*-overexpressing WT (*35S:ABI5-4MYC*) and other related transgenic plants in response to ABA. Seed germination was recorded 2 days after stratification on half-strength MS medium supplemented with 0.5-μM ABA. D, Cotyledon greening of *35S:ABI5-4MYC* and other related transgenic plants in response to ABA. Cotyledon greening was scored 7 days after stratification on half-strength MS medium supplemented with 0.5-μM ABA. Experiments were performed five times by analyzing different batches of seeds. Each batch of seeds of various genotypes was pooled from more than 60 independent plants. For each biological replicate, more than 120 seeds were examined. Values are means ± sd. Bars with different letters are significantly different from each other (*P* < 0.05). Data were analyzed by ANOVA. E, Seedlings of *35S:ABI5-4MYC* and other related transgenic plants 7 days after germination on half-strength MS medium containing 0.5-μM ABA.

To verify that the transcriptional function of ABI5 is enhanced by PRR proteins, we compared the ability of ABI5 to activate downstream targets in mesophyll protoplasts of the wild-type and the *prr5 prr7 prr9* triple mutant. As shown in Figure 9C, *LUC* expression driven by the *EM6* promoter in response to ABA was reduced in *prr5 prr7 prr9* protoplasts compared with its expression in wild-type protoplasts. Similar results were found when the *EM1* promoter was used in these assays (Figure 9C). These findings further support the notion that PRR5 and PRR7 stimulate ABI5’s transcriptional function to modulate downstream genes. Considering that PRR5 and PRR7 interact with ABI5 and enhance its transcriptional function to activate *EM6* and *EM1*, we queried whether PRR proteins directly mediate the expression of *EM6* and *EM1* through binding their promoters. The evidence based on the yeast one-hybrid analysis showed that PRR5 and PRR7 did not recognize the promoter sequences of *EM6* and *EM1* (Supplemental Figures S6 and S7). However, the possibility that PRR proteins are recruited to *EM6* and *EM1* promoters in vivo through interacting with other crucial transcription factors (e.g. ABI5) cannot be ruled out. Previous studies revealed that ABI5 recognizes the *EM6* and *EM1* promoter regions (such as *pEM6-1* and *pEM1-1* shown in Supplemental Table S1) covering a G-box-type cis-element (CACGTG; Carles et al., 2002; Chen et al., 2012). Chromatin immunoprecipitation (ChIP) was performed in ABA-treated germinating seeds of *35S:2FLAG-PRR5-10* and *abi5 35S:2FLAG-PRR5* plants upon ABA treatment. The results showed that PRR5 was enriched at the promoter regions of *EM6* and *EM1* (*pEM6-1* and *pEM1-1*) targeted by ABI5 in *35S:2FLAG-PRR5-10* plants (Figure 10, A and B). However, the enrichment of PRR5 on *pEM6-1* and *pEM1-1* was significantly decreased in *abi5 35S:2FLAG-PRR5* compared with *35S:2FLAG-PRR5-10* (Figure 10, A and B). These findings imply that PRR5 associates with the promoters of *EM6* and *EM1* mainly through ABI5 in vivo.

Hayama et al. (2017) revealed that PRR proteins modulate the stability of their interacting CONSTANS (CO) transcription factor, which promoted us to analyze whether PRR proteins also affects the accumulation of ABI5. The results showed that ABA-induced accumulation of ABI5 was similar in *prr5 prr7 prr9* and wild-type plants (Figure 9D), suggesting that PRR proteins did not regulate the stability of ABI5. As PRR proteins exert stimulative effect on ABI5, we investigated whether the ABA responses of *35S:ABI5-4MYC* were enhanced by the overexpression of *PRR5* during seed germination. To test this possibility, we compared the germination and greening percentages of *35S:ABI5-4MYC* and *35S:ABI5-4MYC/35S:2FLAG-PRR5-10* plants during seed germination in response to ABA. As shown in Figure 10, C–E, the progeny of *35S:ABI5-4MYC/35S:2FLAG-PRR5-10* displayed much lower germination and greening percentages than *35S:ABI5-4MYC*, suggesting that the increased ABA signaling in *35S:ABI5-4MYC* was enhanced by *PRR5* overexpression. The phenotypic observation further supports our proposal that PRR5 stimulates ABI5 to modulate ABA signaling during seed germination.

## Discussion

The circadian clock is an endogenous biological oscillator that modulates a wide range of physiological processes in plants, such as photomorphogenesis, flowering, and stress responses (Yamamoto et al., 2003; FukushiMa et al., 2009; Pruneda-Paz and Kay, 2010; Liu et al., 2013; Atkins and Dodd, 2014; Hsu and Harmer, 2014; Sanchez and Kay, 2016; Frank et al., 2018; Kim et al., 2020; Li et al., 2020a; Simon et al., 2020). The circadian clock also plays crucial roles in the control of ABA biosynthesis and downstream responses (Nováková et al., 2005; Lee et al., 2006; Covington et al., 2008; Mizuno and Yamashino, 2008; FukushiMa et al., 2009; Nakamichi et al., 2009; Penfield and Hall, 2009; McAdam et al., 2011; Seung et al., 2012; Grundy et al., 2015; Adams et al., 2018). However, the detailed mechanisms underlying how ABA signaling is circadian regulated remain elusive. An in-depth understanding of the regulatory effects of central circadian clock components on ABA signaling may help reveal the molecular basis of circadian-mediated ABA signaling. The bZIP-type ABI5 transcription factor is a master regulator of ABA signaling that represses seed germination and early seedling growth (Finkelstein, 1994; Finkelstein and Lynch, 2000a; Lopez-Molina and Chua, 2000; Lopez-Molina et al., 2001, 2002; Brocard et al., 2002; Finkelstein et al., 2005; Skubacz et al., 2016). ABI5 also functions as a critical node to integrate multiple signaling pathways during seed germination and/or postgerminative growth (Lim et al., 2013; Yu et al., 2015; Kim et al., 2016; Hu et al., 2019; Ju et al., 2019; Pan et al., 2020). Despite recent advances, the direct involvement of ABI5 in circadian-modulated ABA responses and the underlying molecular mechanisms are largely unknown.

In this study, we showed that ABI5 physically interacts with PRR5 and PRR7 (Figure 1, A–C), two core proteins of the circadian clock (Yamamoto et al., 2003; Nakamichi et al., 2005, 2010; Farré and Liu, 2013). The interaction between ABI5 and PRR5 or PRR7 was specific because ABI5 did not associate with close homologs of PRR5 and PRR7, such as PRR9 and TOC1 (Figure 1; Supplemental Figure S1). In addition, no interaction was detected between PRR5 or PRR7 and other critical modulators of ABA signaling, such as ABI3 and ABI4 (Figure 1A). Further analysis showed that the bZIP domain of ABI5 and the C-terminal region of PRR5 are essential for the interaction (Figure 2, A and B). In line with the PRR5–ABI5 and PRR7–ABI5 physical interactions, the phenotypic analysis showed that PRR5 and PRR7 positively modulate ABA responses during seed germination. Similar to seeds of the *abi5* mutant, progeny of the *prr5 prr7* double mutant and *prr5 prr7 prr9* triple mutant were hyposensitive to ABA treatment, with much higher percentages of germination and greening than the seeds of the wild-type (Figure 4, A–4; Footitt et al., 2017). Consistent with this result, PRR5 and PRR7 are positively involved in the expression of several downstream ABA-responsive genes, including *EM6*, *EM1*, *RAB18*, and *RD29B* (Figure 5). Conversely, the overexpression of *PRR5* confers germinating seeds with more sensitivity to ABA compared with the wild-type (Figure 6, A–C). On the basis of these results, we concluded that PRR5 and PRR7 interact with ABI5 to activate ABA signaling during seed germination and subsequent seedling establishment in Arabidopsis.

In addition to PRR5, PRR7, and PRR9 proteins, multiple key components of the circadian clock are essential for modulating ABA signaling and/or seed dormancy (Penfield and Hall, 2009; Footitt et al., 2011, 2017; Finch-Savage and Footitt, 2017; Adams et al., 2018). For instance, LHY and CCA1 recognize the promoter regions of several genes critical for ABA biosynthesis and downstream responses (Adams et al., 2018). Phenotypic analysis showed that the germination of *lhy* mutant was impaired in the presence of ABA, whereas *LHY* overexpression led to increased seed germination (Adams et al., 2018). Moreover, disruption of the clock proteins LHY, CCA1, and GIGANTEA (GI) resulted in germination defects in response to low temperature, alternating temperatures, and dry after-ripening (Penfield and Hall, 2009). Further investigations revealed that the transcript levels of central clock genes, such as *LHY*, *CCA1*, *GI*, *TOC1*, *PRR7*, and *PRR9*, do not oscillate in dry seeds (Penfield and Hall, 2009; Footitt et al., 2017). Those studies collectively showed that clock genes do not function in a circadian context in dry seeds and have crucial roles in the suppression of germination (Penfield and Hall, 2009; Footitt et al., 2011, 2017; Finch-Savage and Footitt, 2017; Figures 4 and 6). Interestingly, the expression of several clock genes displays rhythmic patterns during seed imbibition and the clock is restarted (Zhong et al., 1998; Penfield and Hall, 2009; Footitt et al., 2017). Consistently, we also found that *PRR5*, *PRR7*, and *PRR9*, similar to *ABI5*, are rhythmically expressed and responsive to ABA during seed germination (Figure 3). The expression phase of *ABI5* overlaps with those of *PRR5* and *PRR7*, consistent with their ability to interact with plants when expressed normally (Figures 1 and 3). Interestingly, all analyzed ABA-responsive genes are expressed in the same phase as *ABI5* (Figures 3 and 5). We speculated that protein levels for PRR5/7 and ABI5 may follow a similar pattern as their transcript accumulation, but this depends on possible post-transcriptional regulation. Moreover, *PRR7* transcription maintains high levels in the cold winter months and tracks seed dormancyin the deeply dormant winter annual ecotypeCape Verde Island (Footitt et al., 2013, 2014, 2017). It is possible that PRR7, as well as its close homologs PRR5 and PRR9 functions in the winter months to enhance ABA signaling and suppress seed germination.

Previous studies revealed that PRR5, PRR7, and PRR9 play pivotal roles in multiple clock-associated physiological processes (Yamamoto et al., 2003; Nakamichi et al., 2005, 2007, 2010; Farré and Liu, 2013; Li et al., 2019; Yuan et al., 2021). For instance, PRR5, PRR7, and PRR9 act as transcriptional repressors in the circadian clock and interact with TOPLESS/TOPLESS-RELATED (TPL/TPR) and HISTONE DEACETYLASE6 (HDA6) to restrict the expression of the core clock genes *CCA1* and *LHY* (Nakamichi et al., 2010; Farré and Liu, 2013; Wang et al., 2013; Liu et al., 2016). These three PRR proteins also directly suppress cold-induced expression of *CREPEAT BINDING FACTOR/DRE BINDING FACTOR1* (*CBF*/*DREB1*) genes and negatively modulate freezing tolerance (Nakamichi et al., 2009). Conversely, PRR5, PRR7, and PRR9 stabilize CO to enhance the expression of *FLOWERING LOCUS T* (*FT*) and promote flowering (Nakamichi et al., 2007; Hayama et al., 2017). PRR9 directly activates transcription of *ORESARA1* (*ORE1*) and positively regulates leaf senescence (Kim et al., 2018). Our data show that PRR5 and PRR7 stimulate the transcriptional function of ABI5 to upregulate ABA-induced expression of *EM6* and *EM1* (Figure 9, B and C). PRR5 also associates with the *EM6* and *EM1* promoters mainly through ABI5 (Figure 10, A and B). Further phenotypic analysis found that the overexpression of *ABI5* and *PRR5* simultaneously confers plants much more sensitive to ABA during seed germination compared with the overexpression of *ABI5* alone (Figure 10, C–E). These results collectively demonstrate that PRR5 is a positive modulator of ABI5-mediated signaling during seed germination. Given that the bZIP domain required for dimerization and DNA binding of ABI5 is involved in the interaction with PRR5 (Figure 2A), it is perhaps surprising that addition of PRR5 enhances rather than inhibits ABI5 function. As PRR5 is recruited to *EM6* and *EM1* promoters in vivo through interacting with ABI5 (Figure 10, A and B), it is possible that the PRR5–ABI5 complex may function similarly as the dimers of ABI5 and have increased binding activity on promoters of target genes (e.g. *EM6* and *EM1*). In addition, PRR5 may compete with some repressors of ABI5 to bind the bZIP domain and interfere with the regulatory effects of those repressors. Nevertheless, the detailed biochemical mechanisms underlying how these PRR proteins synergize with ABI5 to modulate downstream genes deserve further investigation. Because these PRR proteins could have dual regulatory effects (negative or positive) on their targets and/or interacting partners, they may help to establish an appropriate balance among different development- or stress-signaling pathways so that growth and stress tolerance are optimized for the prevailing conditions.

Genetic analysis found that the progeny of *abi5 35S:2FLAG-PRR5*, similar to the *abi5* seeds, was also hyposensitive to ABA treatment compared with those of the wild-type (Figure 7, A– C). This result demonstrates that the increased ABA signaling in *PRR5*-overexpressing plants requires ABI5. However, the possibility that PRR5 and PRR7 associate with other proteins to modulate ABA responses during seed germination cannot be ruled out. As shown in Figure 7, A–C, although the *abi5 35S:2FLAG-PRR5* plants mimicked the phenotype of *abi5*, the performances of *abi5 35S:2FLAG-PRR5* and *abi5* were significantly different. Consistent with this notion, the *prr5 prr7 abi5* triple mutant exhibited higher germination and greening percentages than *abi5* and *prr5 prr7* in the presence of ABA (Figure 8, A–C). ABI3 and ABI4 also are crucial transcriptional regulators of ABA signaling that are involved in repressing seed germination (Giraudat et al., 1992; Finkelstein, 1994; Finkelstein et al., 1998; Suzuki et al., 2001). However, no physical interaction between PRR5 or PRR7 and ABI3 or ABI4 was detected in yeast (Figure 1A). *EM6* and *EM1* are direct downstream target genes of ABI5 (Carles et al., 2002). The yeast one-hybrid screening found that PRR5 and PRR7 did not bind the promoter sequence of *EM6* and *EM1* (Supplemental Figures S6 and S7).These observations imply that PRR5 and PRR7 may not directly interact with ABI3, ABI4, EM6, and EM1 in ABA signaling. Nevertheless, ChIP assays showed that PRR5 may associate indirectly with the promoters of *EM6* and *EM1* through ABI5 (Figure 10, A and B). Further elucidation of potential associations of PRR5 and PRR7 with other key regulators of ABA responses will further enhance our understanding of PRR5- and PRR7-mediated ABA signaling networks.

**Figure 11:**
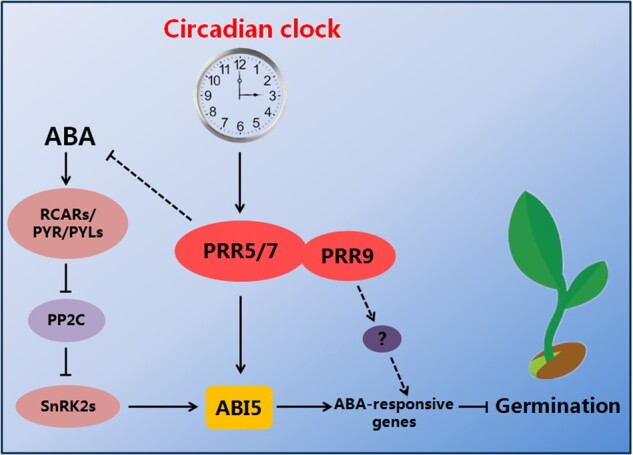
Simplified model for the interactions of ABI5 and PRR proteins in modulating ABA signaling during seed germination. When the concentration of ABA is elevated, ABA induces the expression of *ABI5* as well as *PRR5*, *PRR7*, and *PRR9* during seed germination. PRR5 and PRR7 physically interact with ABI5 and stimulate its transcriptional function to enhance ABA signaling and maintain proper seed germination and postgerminative growth. In addition, PRR5, PRR7, and PRR9 are negatively involved in ABA biosynthesis.

Our phenotypic investigation showed that seeds of the *prr5 prr7 prr9* triple mutant had much higher germination and greening percentages than seeds of *prr5 prr7* and *prr5 prr9* double mutants in response to ABA (Figure 4, A–C). This observation suggests that PRR9 may act together with PRR5 and PRR7 to positively modulate ABA responses during seed germination. However, unlike PRR5 and PRR7, PRR9 did not interact with ABI5 to form a protein complex (Figure 1, A and B), implying that PRR9 is not involved directly in ABI5-mediated ABA signaling through a PRR9–ABI5 interaction during seed germination. It is possible that PRR9 may function with PRR5 and PRR7 to mediate ABA responses via other modulators in ABA signaling. To better understand the molecular mechanism of the core circadian clock proteins PRR5/7/9-regulated ABA signaling in Arabidopsis, we constructed the simplified model involving PRR5/7/9 and ABI5 shown in Figure 11. When the concentration of ABA is elevated, ABA induces the expression of ABI5 as well as PRR5/7/9 during early stage of seed germination. PRR5 and PRR7 physically interact with ABI5 and stimulate its transcriptional function to enhance ABA signaling and maintain proper seed germination and postgerminative growth. In addition, PRR5, PRR7, and PRR9 may modulate ABA responses through other components of ABA signaling and negatively involve in ABA biosynthesis. Taken together with the fact that the transcript levels of *ABI5* displayed a circadian pattern in response to ABA (Figure 3), it is possible that circadian clock exhibits dual regulatory effects (at transcriptional level and protein level) on ABI5-mediated ABA signaling during seed germination. These dual regulations of ABI5-mediated ABA signaling by circadian clock may be adaptive mechanisms to establish appropriate ABA signaling during seed germination.

Zhao et al. (2019) reported that the brassinosteroid-related BES1 transcription factor physically associates with ABI5 and attenuates its transcriptional activity, thereby integrating brassinosteroid and ABA signals to modulate seed germination. In a previous study, we found that two proteins of the VQ family, VQ18 and VQ26, interact with ABI5 to form a complex (Pan et al., 2018). We also showed that VQ18 and VQ26 interfere with the transcriptional function of ABI5 to negatively mediate ABA signaling during seed germination and postgerminative growth. Chen et al. (2012) found that the Arabidopsis mediator subunit MEDIATOR 25 (MED25) directly bind ABI5 and represses ABA responses. MED25 affects the stability of ABI5 as well as the recruitment of ABI5 to the promoter sequences of its target genes (Chen et al., 2012; Guo et al., 2021). Conversely, Lim et al. (2013) showed that DELLA proteins interact with ABI5 to upregulate the expression of a subset of high temperature-inducible genes and suppress seed germination. Kim et al. (2016) found that the PHYTOCHROME-INTERACTING FACTOR 1 (PIF1) transcription factor functions together with ABI5 to bind the promoters of downstream target genes. Together, these results and our present findings suggest that the distinct regulatory effects of these interacting factors on ABI5 may be specific adaptive mechanisms to integrate diverse signals and establish appropriate ABA signaling levels, thereby ensuring efficient stress tolerance while minimizing the detrimental effect of ABA on germination and early seedling growth.

## Materials and methods

### Materials and plant growth conditions

Taq DNA polymerases were obtained from Takara Biotechnology (Dalian, China), and other common chemicals were purchased from Shanghai Sangon (Shanghai, China). The phytohormone ABA was purchased from Sigma-Aldrich. The wild-type and mutant *A.* *thaliana* plants used in this study were in the Columbia (Col-0) genetic background. The *prr5-1* (SALK_006280), *prr5-2* (SALK_135000C), *prr7-1* (SALK_091569C), and *prr7-2* (SALK_030430C) mutants were obtained from the Arabidopsis Resource Center at Ohio State University (http://abrc.osu.edu). The *prr5 prr7* double mutant was generated by genetically crossing *prr5-1* with *prr7-2* using standard techniques. Seeds of *prr5-1 prr9-1* (*prr5 prr9*) and *prr5-1 prr7-2 prr9-1* (*prr5 prr7 prr9*; Li et al., 2019) were provided by Prof. Lei Wang (Institute of Botany, Chinese Academy of Sciences). The transgenic line *35S:ABI5-4MYC* (Chen et al., 2012) was provided by Prof. Chuanyou Li (Institute of Genetics and Developmental Biology, Chinese Academy of Sciences). To generate *35S:2FLAG-PRR5* transgenic plants, the full-length cDNAs of *PRR5* behind the 2FLAG tag sequences were cloned into the binary vector pOCA30 in the sense orientation behind the CaMV 35S promoter (Hu et al., 2013).The Arabidopsis plants were grown in an artificial growth chamber at 22°C under a 16-h-light (100-µE m^−2^s^−1^, white fluorescent bulbs, full-spectrum light), 8-h-dark photoperiod.

### Yeast two-hybrid assays

The full-length CDS of *CCA1*, *LHY*, *PRR9*, *PRR7*, *PRR5*, *PRR3*, and *TOC1* were fused to pGBKT7 (Clontech) to generate bait vectors (BD-CCA1, BD-LHY, BD-PRR, and BD-TOC1) that contain the Gal4 DNA-BD. Full-length CDS of *ABI5*, *ABI4*, and *ABI3* were inserted into pGADT7 (Clontech) to produce prey vectors (AD-ABI) with the Gal4 AD. To identify specific regions critical for the interactions, multiple truncated PRR5 sequences were fused to pGBKT7 and truncated ABI5 sequences were ligated with pGADT7. Yeast two-hybrid assays were performed as described previously (Hu et al., 2013). The bait and prey vectors were cotransformed into the yeast strain AH109 and physical interactions were indicated by the ability of cells to grow on dropout medium lacking Leu, Trp, His, and Ade for 4 days after plating. The primers used for cloning are listed in Supplemental Data Set S1.

### BiFC assays

The cDNA sequences encoding the C-terminal 64-amino acid enhanced YFP (cYFP) fragments and N-terminal 173-amino acid YFP (nYFP) were PCR amplified and individually inserted into tagging pFGC5941 plasmids to produce pFGC-cYFP and pFGC-nYFP, respectively (Kim et al., 2008). Full-length cDNA or the sequences encoding the 164 N-terminal residues of ABI5 were cloned into pFGC-cYFP to produce a C-terminal in-frame fusion with cYFP (ABI5-cYFP or ABI5^1–164^-cYFP). Full-length PRR5, PRR7, and PRR9 were inserted into pFGC-nYFP to generate an N-terminal in-frame fusion with nYFP (PRR5-nYFP, PRR7-nYFP, and PRR9-nYFP). The resulting plasmids were transformed into *Agrobacterium tumefaciens* strain GV3101, and infiltration of wild tobacco (*N.* *benthamiana*) leaves was performed at zeitgeber time 12 as described previously (Hu et al., 2019). Infected leaves with YFP and DAPI fluorescence were detected 40–52 h after infiltration under a confocal laser-scanning microscope (Olympus, Tokyo, Japan). The experiments were performed at least four times using different batches of wild tobacco plants; for each biological replicate, more than 12 tobacco plants were infiltrated and more than 600 cells were analyzed. The primers used for cloning are listed in Supplemental Data Set S1.

### CoIP assays

To confirm the ABI5–PRR5 interaction, whole proteins were extracted from samples harvested at ZT12 of 0.5-μM ABA-treated (for 2.5 days) germinating seeds of transgenic Arabidopsis simultaneously overexpressing *ABI5* and *PRR5* (*35S:ABI5-4MYC/35S:2FLAG-PRR5*), which was constructed by introducing *PRR5* overexpression (*35S:2FLAG-PRR5*) into previously described *35S:ABI5-4MYC* plants (Chen et al., 2012; Hu et al., 2019). Total proteins were prepared from Arabidopsis plants with an extraction buffer containing 50-mM Tris–HCl (pH 7.4), 1-mM EDTA, 150-mM NaCl, 10% (v/v) glycerol, 0.1% (v/v) Triton X-100, 1-mM PMSF, and 1x Roche Protease Inhibitor Cocktail. Immunoprecipitation experiments were performed with protein A/G Plus-agarose beads following the manufacturer’s protocol. In brief, cell lysates were precleared with the protein A/G Plus-agarose beads and incubated with the anti-MYC antibody (catalog no. A7470, Sigma-Aldrich; 1:250) and the protein A/G Plus-agarose beads at 4°C overnight in the extraction buffer. The beads were washed twice extensively with the extraction buffer and the co-immunoprecipitated protein was then detected by immunoblotting using an anti-FLAG antibody (catalog no. F7425, Sigma-Aldrich; 1:10,000).

### Determination of germination and greening

The germination and greening of the wild-type and mutant seeds were determined as described previously (Hu et al., 2019). Briefly, seeds were first hydrated at ZT0, sown on medium with or without supplementation of ABA, and cold stratified at 4°C/dark for 4 days. Then, they were transferred at ZT0 to an artificial growth chamber at 22°C under 16-h light and 8-h-dark conditions for germination. Germination was determined based on the appearance of the embryonic axis (i.e. radicle protrusion) as observed under a microscope. Seedling greening was determined based on the appearance of green cotyledons on seedlings. To analyze the ABA sensitivity of germination and greening, seeds were plated on water agar (0.6%) medium or half-strength MS medium supplemented with ABA. More than three independent experiments were performed, and similar results were obtained.

### RNA extraction and RT-qPCR

Total RNA was extracted from germinating seeds (2 days, harvested from ZT0 to ZT36) of the wild-type and/or *prr5 prr7 prr9* with or without 0.5-μM ABA treatment using the Trizol reagent (Invitrogen) and RT-qPCR was performed as described previously (Han et al., 2020). Briefly, 1.0-μg DNase-treated RNA was reverse-transcribed in a 20-μL reaction volume with oligo (dT)_18_ primer using Moloney murine leukemia virus reverse transcriptase (Fermentas). Then, 1.0-μL cDNA was used for RT-qPCR with the SYBR Premix Ex Taq kit (Takara) on a Roche LightCycler 480 real-time PCR machine, according to the manufacturer’s instructions. At least three biological replicates for each sample were used for RT-qPCR analysis. The *At1g13320* gene, which encodes a subunit of Ser/Thr PP2A and is stably expressed in seed samples during germination (Czechowski et al., 2005), was used as the control. The gene-specific primers used for the RT-qPCR are listed in Supplemental Data Set S1.

### Transient transactivation assays

Full-length *ABI5*, *PRR5*, *PRR7*, and *GFP* sequences were PCR amplified and cloned into the pGreenII 62-SK vector as effectors (Hellens et al., 2005). The putative promoter sequences of *EM1* (2,000 bp) and *EM6* (1273 bp) were amplified and fused to the pGreenII 0800-LUC vector as reporters (Hellens et al., 2005). Combinations of plasmids were transformed into the wild-type or *prr5 prr7 prr9* mutant Arabidopsis leaf mesophyll protoplasts according to the Sheen laboratory protocol (Sheen, 2001). Transfected cells were cultured for 10–16 h with or without 5-μM ABA treatment, and the relative LUC activity was analyzed using a Dual-Luciferase Reporter Assay system (Promega, Madison, WI, USA), which measured the activities of firefly LUC and the internal control *Renillareniformis* LUC (REN). The primers used for cloning are listed in Supplemental Data Set S1.

### Yeast one-hybrid assays

The yeast one-hybrid assays were performed using the Matchmaker Yeast One-Hybrid System Kit (Clontech) according to the manufacturer’s instructions. Full-length CDS of *PRR5* and *PRR7* were inserted into pGADT7 to produce AD-PRR constructs. The putative promoter fragments of *EM1* and *EM6* were cloned into the pAbAi vector to generate pAbAi-pEM1 and pAbAi-pEM6, which were linearized by BstBI, and then transformed into the Y1HGold yeast strain. The transformed cells were grown in the SD/-Ura plate for 3 days. AD-PRR5 and AD-PRR7 were then transformed into the strain harboring pAbAi-pEM1 or pAbAi-pEM6 and selected on the SD/-Leu plate. Cotransformed cells were cultured on an SD/-Leu plate containing aureobasidin A (AbA, 200 µg·L^-1^) for 3 days, and positive clones were spotted in several yeast concentrations from dilution of 10° (OD_600_ = 1.0) to 10^−3^. The primers used for cloning are listed in Supplemental Data Set S1.

### ChIP assays

The ChIP assay was performed essentially as described previously (Mukhopadhyay et al., 2008; Jiang et al., 2014). Briefly, germinating seeds (with or without 0.5-μM ABA treatment for 2.5 days; harvested at ZT12) of the wild-type, *35S:2FLAG-PRR5-10*, and *abi535S:2FLAG-PRR5* were cross-linked in 1% formaldehyde and their chromatin isolated. The anti-FLAG antibody was used to immunoprecipitate the protein–DNA complex, and the precipitated DNA was purified using a PCR purification kit (Qiagen) for qPCR analysis. To quantitatively determine the PRR5–DNA (target promoter) binding, qPCR analysis was performed according to the procedure described previously (Mukhopadhyay et al., 2008) with the promoter sequence of *PP2A* (At1g13320) gene as an endogenous control. The relative quantity value was calculated by the 2 (^–DD^^*C*^^t^) method (Mukhopadhyay et al., 2008) and presented as the DNA binding ratio. The qPCR data from ChIPassay with anti-FLAG antibody with the *PP2A* (At1g13320) promoter as a negative control. The results shown were obtained from three biological replicates using different batches of seeds. The primers used for ChIP assays are listed in Supplemental Data Set S1.

### Statistical analysis

Statistical analysis was performed by analysis of variance. The results are shown in Supplemental Table S2.

### Accession numbers

The genes discussed in this article can be found in the Arabidopsis Genome Initiative database as follows: *ABI5*, AT2G36270; *ABI4*, AT2G40220; *ABI3*, AT3G24650; *PRR5*, AT5G24470; *PRR7*, AT5G02810; *PRR9*, AT2G46790;*PRR3*, AT5G60100; *TOC1*, AT5G61380; *LHY*, AT1G01060; *CCA1*, AT2G46830; *EM1*, AT3G51810; *EM6*, AT2G40170; *RAB18*, AT1G43890; and *RD29B*, AT5G52300.

## Supplemental data 

The following materials are available in the online version of this article.

**Supplemental Figure S1**. Yeast two-hybrid assay analysis of the interactions of ABI5 with PRR5, PRR3, TOC1, LHY, and CCA1 proteins.

**Supplemental Figure S2**. ABA responses of *prr5* and *prr7* single mutants during seed germination.

**Supplemental Figure S3**. ABA responses of *prr5 prr7*, *prr5 prr9*, and *prr5 prr7 prr9* mutants during seed germination on water agar medium.

**Supplemental Figure S4**. RT-qPCR analysis of *PRR5* expression in overexpression lines.

**Supplemental Figure S5**. ABA responses of *PRR5*-overexpressing plants during seed germination on water agar medium.

**Supplemental Figure S6**. Yeast one-hybrid assay on binding of PRR5 and PRR7 to the promoter region of *EM6*.

**Supplemental Figure S7**. Yeast one-hybrid assay on binding of PRR5 and PRR7 to the promoter region of *EM1*.

**Supplemental Table S1**. Information for ABI5-binding promoter sequences of *EM6* and *EM1* (*pEM6-1* and *pEM1-1*).

**Supplemental Table S2**. Analysis of variance (ANOVA) tables.

**Supplemental Data Set S1**. Primers used for cloning, RT-qPCR, and ChIP analysis.

## Supplementary Material

koab168_Supplementary_Data

## Notes

Y.H. and M.Y. designed this study and wrote the article; M.Y., X.H., J.Y., Y.J., and Y.H. performed experiments or interpreted data; all authors approved the final article.

The author responsible for distribution of materials integral to the findings presented in this article in accordance with the policy described in the Instructions for Authors (https://academic.oup.com/plcell) is: Yanru Hu (huyanru@xtbg.ac.cn).
