# Supplementary material for: The Arabidopsis circadian clock protein PRR5 interacts with and stimulates ABI5 to modulate abscisic acid signaling during seed germination
Source: Plant Cell. 2021 Jun 21;33(9):3022–41. doi: 10.1093/plcell/koab168 (PMC8462813; doi:10.1093/plcell/koab168)
Supplement: koab168_Supplementary_Data [file koab168_supplementary_data.zip › tpc.00998.2020-s01.pdf]

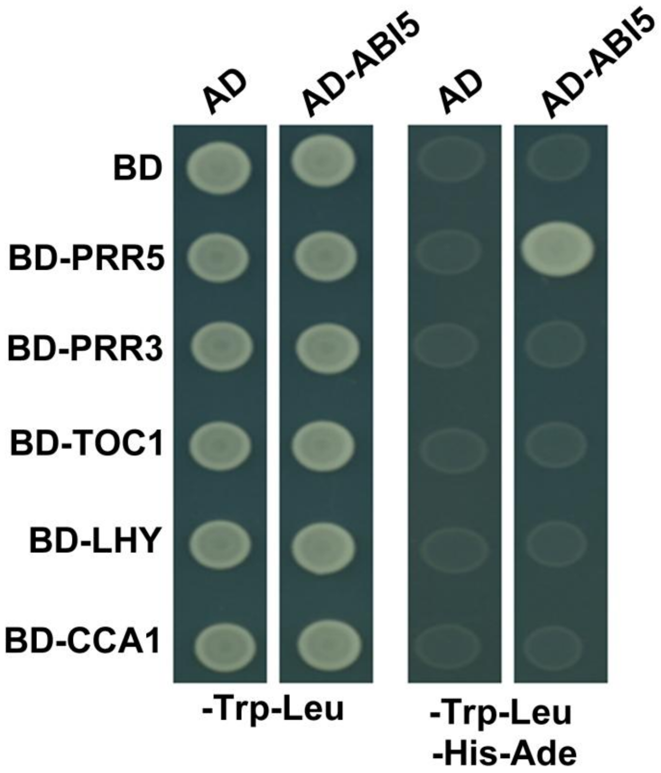

Supplemental Figure S1. Yeast Two-Hybrid Assay Analysis of the Interactions of ABI5 with PRR5, PRR3, TOC1, LHY, and CCA1 Proteins. (Supports Figure 1).

Interaction is indicated by the ability of yeast cells to grow on dropout medium lacking Leu, Trp, His, and Ade for 4 d after plating. pGBKT7 (BD) and pGADT7 (AD) were used as negative controls.

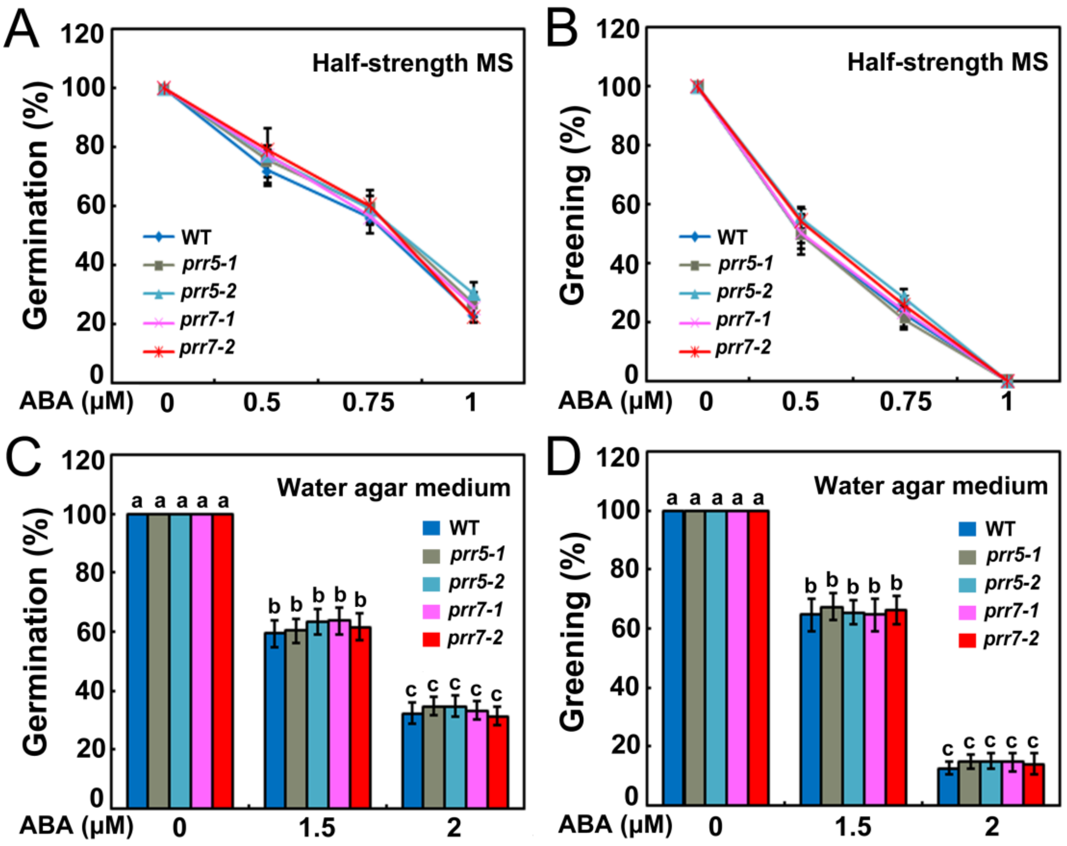

Supplemental Figure S2. ABA Responses of *prr5* and *prr7* Single Mutants during Seed Germination. (Supports Figure 4).

(A) Germination of the wild type (WT), *prr5,* and *prr7* single mutants in response to ABA on half-strength Murashige and Skoog (MS) medium. Seed germination was recorded 2 d after stratification on half-strength MS medium supplemented with different concentrations of ABA.

(B) Cotyledon greening of the WT, *prr5,* and *prr7* mutants in response to ABA on half-strength MS medium. Cotyledon greening was scored 5 d after stratification on half-strength MS medium supplemented with different concentrations of ABA.

(C) Germination of the WT, *prr5,* and *prr7* single mutants in response to ABA on water agar medium. Seed germination was recorded 1.5 d after stratification on water agar medium supplemented with different concentrations of ABA.

(D) Cotyledon greening of the WT, *prr5,* and *prr7* mutants in response to ABA on water agar medium. Cotyledon greening was scored 4 d after stratification on water agar medium supplemented with different concentrations of ABA.

Experiments described above were performed three times by analyzing different batches of seeds. Each batch of seeds of WT, *prr5,* and *prr7* single mutants was pooled from more than 60 independent plants. For each biological replicate, more than 120 seeds were examined. Values are means ± SD. Bars with different letters are significantly different from each other ($P < 0.05$). Data were analyzed by ANOVA.

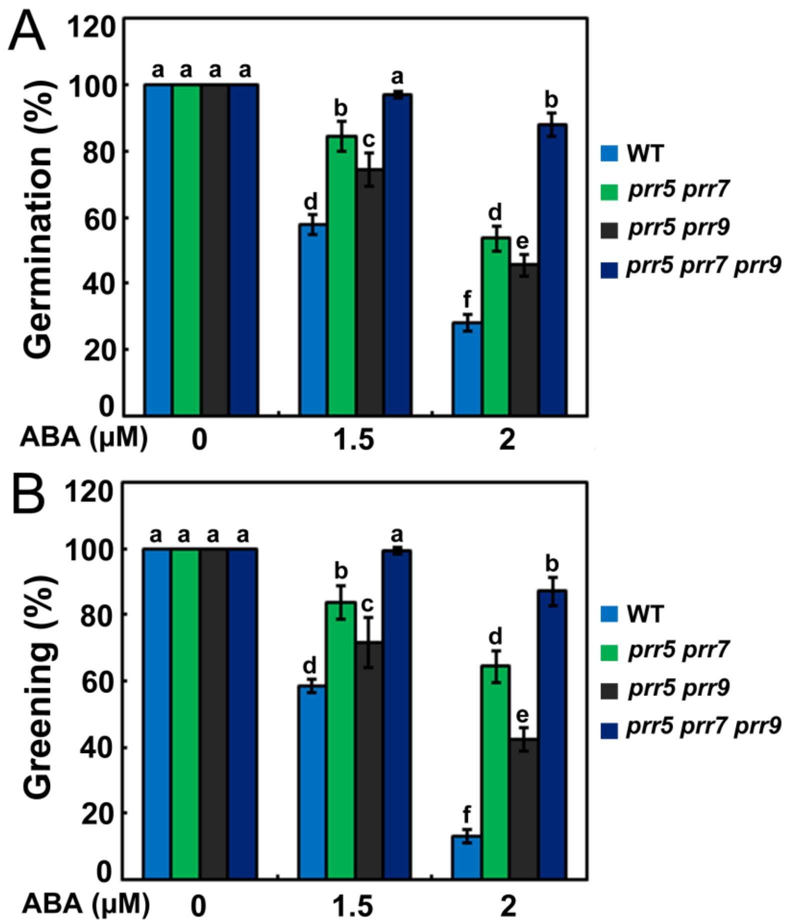

Supplemental Figure S3. ABA Responses of *prr5 prr7*, *prr5 prr9*, and *prr5 prr7 prr9* Mutants during Seed Germination on Water Agar Medium. (Supports Figure 4).

(A) Germination of the wild type (WT), *prr5 prr7*, *prr5 prr9*, and *prr5 prr7 prr9* mutants in response to ABA on water agar medium. Seed germination was recorded 1.5 d after stratification on water agar medium supplemented with different concentrations of ABA.

(B) Cotyledon greening of the WT, *prr5 prr7*, *prr5 prr9*, and *prr5 prr7 prr9* mutants in response to ABA on water agar medium. Cotyledon greening was scored 4 d after stratification on water agar medium supplemented with different concentrations of ABA.

Experiments described above were performed three times by analyzing different batches of seeds. Each batch of seeds of WT, *prr5 prr7*, *prr5 prr9*, and *prr5 prr7 prr9* mutants was pooled from more than 60 independent plants. For each biological replicate, more than 120 seeds were examined. Values are means ± SD. Bars with different letters are significantly different from each other ($P < 0.05$). Data were analyzed by ANOVA.

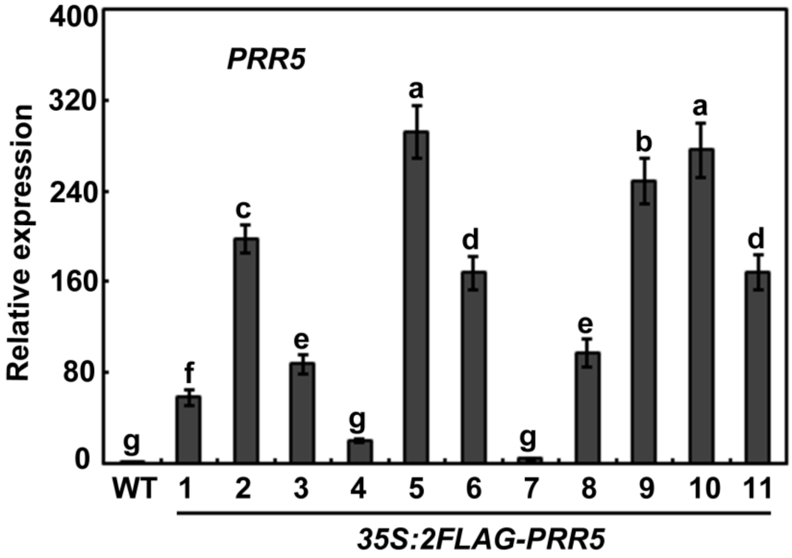

Supplemental Figure S4. RT-qPCR Analysis of *PRR5* Expression in Overexpression Lines. (Supports Figure 6).

Total RNA was extracted from three different batches of germinating seeds (3 d) of the wild type (WT) and *PRR5*-overexpressing plants (*35S:2FLAG-PRR5*) as biological replicates. The *PP2A* (AT1G13320) gene was used as control. Error bars show SD from three independent biological replicates. Values are means ±SD. Bars with different letters are significantly different from each other ($P < 0.05$). Data were analyzed by ANOVA.

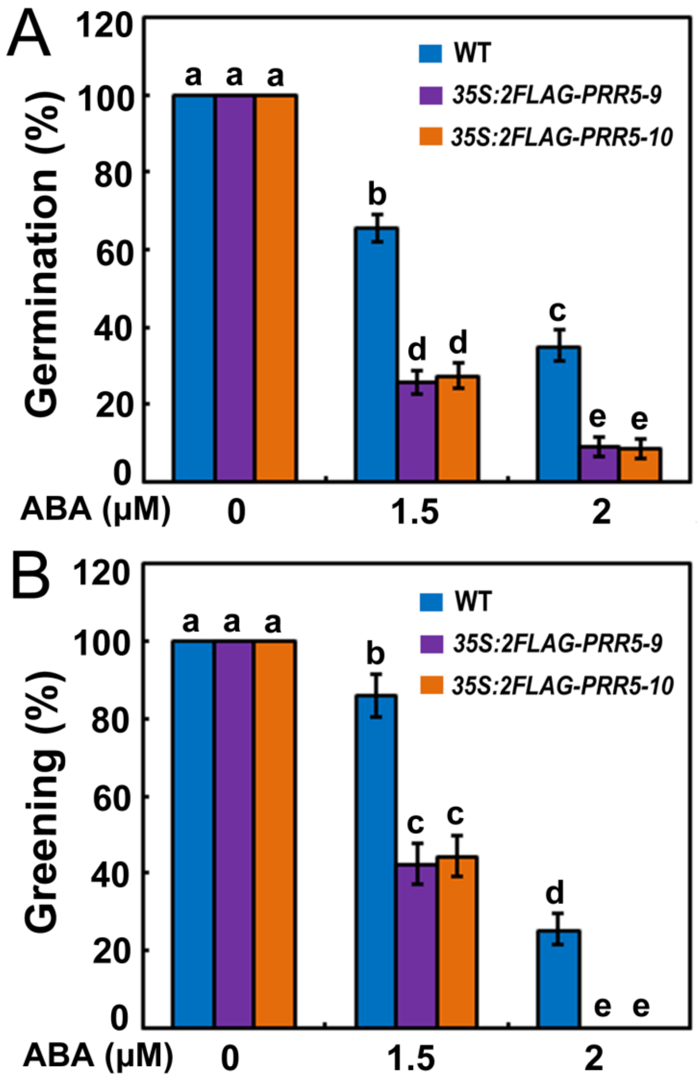

Supplemental Figure S5. ABA Responses of *PRR5*-Overexpressing Plants during Seed Germination on Water Agar Medium. (Supports Figure 6).

(A) Germination of the wild type (WT) and *PRR5*-overexpressing plants *35S:2FLAG-PRR5-9* and *35S:2FLAG-PRR5-10* in response to ABA on water agar medium. Seed germination was recorded 1.5 d after stratification on water agar medium supplemented with different concentrations of ABA.

(B) Cotyledon greening of the WT, *35S:2FLAG-PRR5-9,* and *35S:2FLAG-PRR5-10* in response to ABA on water agar medium. Cotyledon greening was scored 5 d after stratification on water agar medium supplemented with different concentrations of ABA.

Experiments described above were performed three times by analyzing different batches of seeds. Each batch of seeds of WT, *35S:2FLAG-PRR5-9,* and *35S:2FLAG-PRR5-10* was pooled from more than 60 independent plants. For each biological replicate, more than 120 seeds were examined. Values are means ± SD. Bars with different letters are significantly different from each other ($P < 0.05$). Data were analyzed by ANOVA.

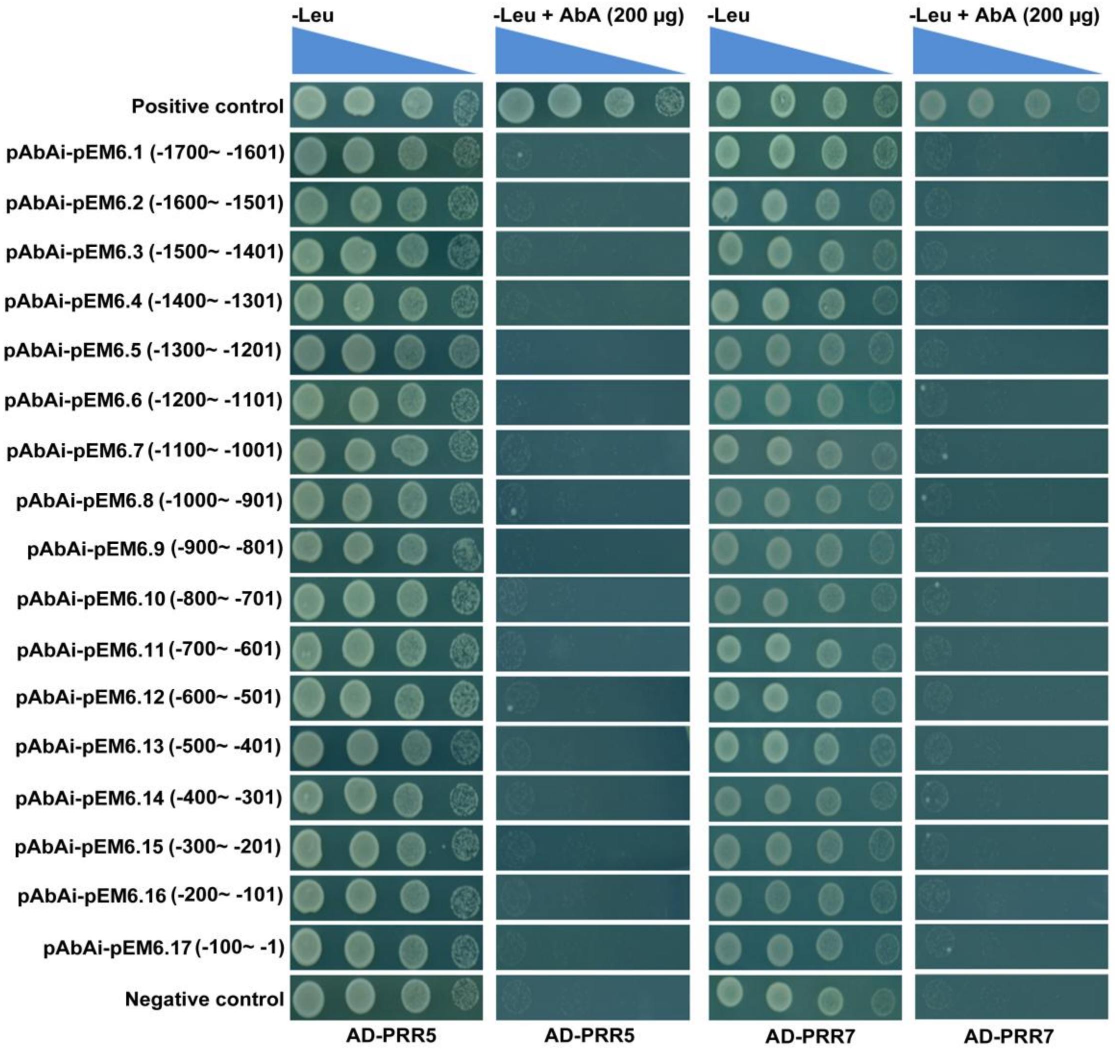

Supplemental Figure S6. Yeast One-Hybrid Assay on Binding of PRR5 and PRR7 to the Promoter Region of *EM6.* (Supports Figure 10).

Yeast one-hybrid assay on binding of PRR5 and PRR7 to the promoter regions of *EM6. pEM6.1* to *pEM6.17* represent different regions of the *EM6* promoter, and the fragment location site is relative to the start code ATG. Blue triangle represents the range of yeast concentrations from dilution of 10^0 (OD_{600}=1.0) to 10^{-3}. pGADT7-p53+pAbAi-p53 was used as a positive control and pGADT7+pAbAi-p53 as a negative control.

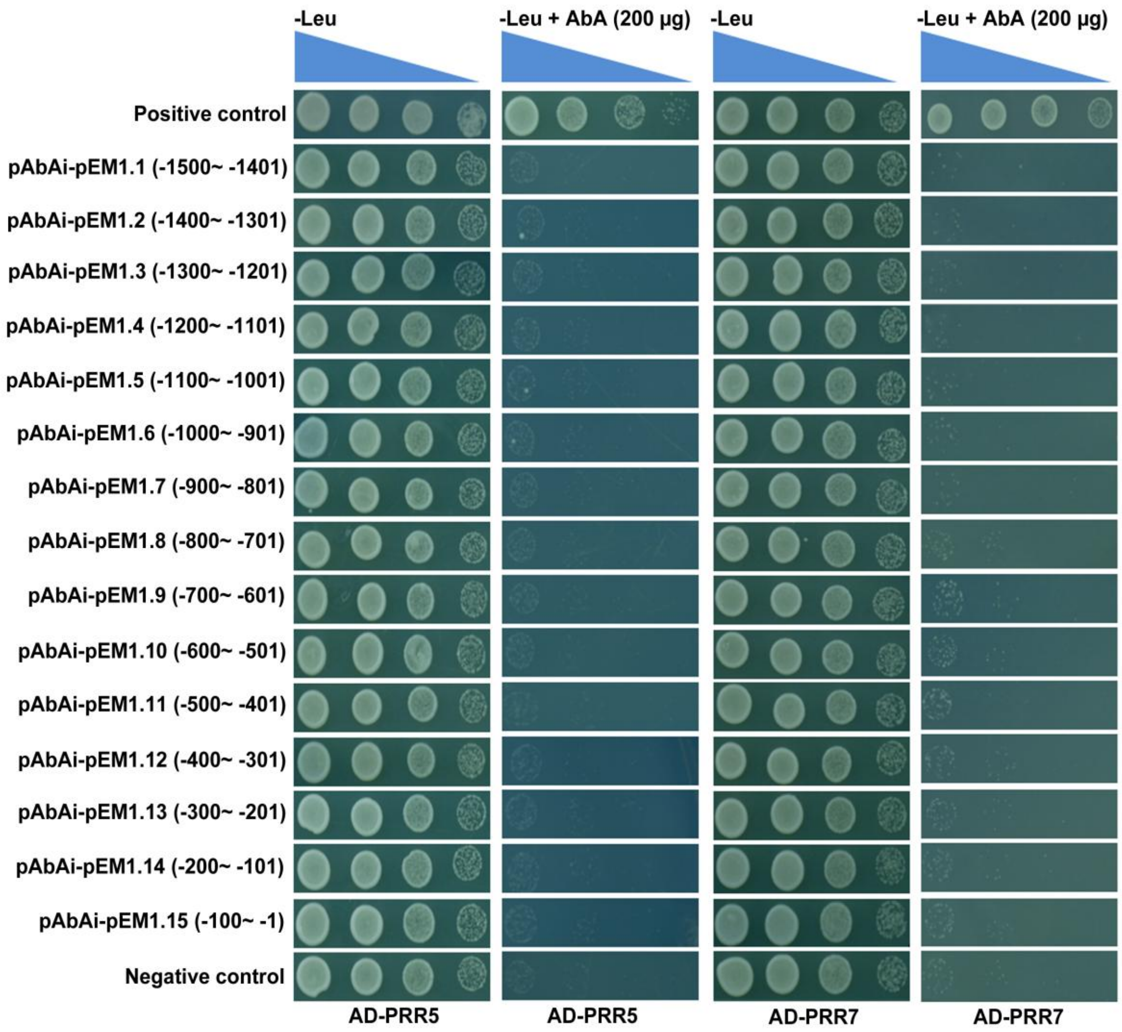

Supplemental Figure S7. Yeast One-Hybrid Assay on Binding of PRR5 and PRR7 to the Promoter Region of *EM1*. (Supports Figure 10).

Yeast one-hybrid assay on binding of PRR5 and PRR7 to the promoter regions of *EM1. pEM1.1* to *pEM1.15* represent different regions of the *EM1* promoter, and the fragment location site is relative to the start code ATG. Blue triangle represents the range of yeast concentrations from dilution of 10^0 (OD_{600}=1.0) to 10^{-3}. pGADT7-p53+pAbAi-p53 was used as a positive control and pGADT7+pAbAi-p53 as a negative control.

Supplemental Table S1. Information for ABI5-binding promoter sequences of *EM6* and *EM1 (pEM6-1* and *pEM1-1).* In promoter fragment names, prefix 'p' indicates promoter. Location, the fragment location site relative to the start code ATG.

pEM6-1:

Location: -272 ~ -73; Sequences (200 bp):

CACGTTCAAAGTATACGTCAGCTAATAAAGTCAGACACGTGGCATGTCACCAAGAAA
GAAGAGCATACGTATGACGTACCGATTGTCCCTGAGTCACCACGTCGAACATCCACG
ATGCAACACAACCAAAACGCCCTCCGGATTGAACTCGCTATATATAGAGCGAGCTAC
TATAGTCTTCTTCGTATATCATCAAGTCG

pEM1-1:

Location: -199 ~ -14; Sequences (185 bp):

GAGTCGTGTCAAGCAGCTCGTTAATAACTGTAGCAAGTTGACTGAGTAAGCATCAAC
GTGTCATCTCCGTAAAGCCCATTATTTCTAGTCTCGCCGCGTCTTCTCTTCCACGTA
GCACTTCACTTTTTCTCTCCTTTTGTTTCCTTTGGAACACAAACGTTTCTATTTATA
GGAATAATTACGTC

Supplemental Table S2. ANOVA tables

Sum Sq = Sum of squares; df = degrees of freedom; Mean Sq = Mean Squares

Figure 6B

	df	Sum Sq	Mean Sq	F-value	P-value
Treatment	8	85434.438	10679.305	2643.277	< 0.0001
Residual	36	145.446	4.040		
Total	44	85579.885			

Figure 7A

	df	Sum Sq	Mean Sq	F-value	P-value
Treatment	7	32154.162	4593.452	476.709	< 0.0001
Residual	32	308.344	9.636		
Total	39	32462.506			

Figure 7B

	df	Sum Sq	Mean Sq	F-value	P-value
Treatment	7	48070.032	6867.147	1531.358	< 0.0001
Residual	32	143.499	4.484		
Total	39	48213.531			

Figure 8A

	df	Sum Sq	Mean Sq	F-value	P-value
Treatment	7	25250.711	3607.244	521.765	< 0.0001
Residual	32	221.234	6.914		
Total	39	25471.945			

Supplemental Table S2. ANOVA tables

Sum Sq = Sum of squares; df = degrees of freedom; Mean Sq = Mean Squares

Figure 8B

	df	Sum Sq	Mean Sq	F-value	P-value
Treatment	7	43437.242	6205.320	1178.010	< 0.0001
Residual	32	168.564	5.268		
Total	39	43605.806			

Figure 9B (for promoter *EM6*)

	df	Sum Sq	Mean Sq	F-value	P-value
Treatment	13	1301507.891	100115.992	271.814	< 0.0001
Residual	28	10313.093	368.325		
Total	41	1311820.985			

Figure 9B (for promoter *EM1*)

	df	Sum Sq	Mean Sq	F-value	P-value
Treatment	13	738223.444	56786.419	358.590	< 0.0001
Residual	28	4434.087	158.360		
Total	41	742657.531			

Figure 9C (for promoter *EM6*)

	df	Sum Sq	Mean Sq	F-value	P-value
Treatment	7	308212.205	44030.315	318.496	< 0.0001
Residual	16	2211.913	138.245		
Total	23	310424.118			

Supplemental Table S2. ANOVA tables

Sum Sq = Sum of squares; df = degrees of freedom; Mean Sq = Mean Squares

Figure 9C (for promoter *EM1*)

	df	Sum Sq	Mean Sq	F-value	P-value
Treatment	7	192937.958	27562.565	256.217	< 0.0001
Residual	16	1721.200	107.575		
Total	23	194659.158			

Figure10A

	df	Sum Sq	Mean Sq	F-value	P-value
Treatment	11	96.088	8.735	121.179	< 0.0001
Residual	24	1.730	0.072		
Total	35	97.819			

Figure 10B

	df	Sum Sq	Mean Sq	F-value	P-value
Treatment	11	51.516	4.683	108.014	< 0.0001
Residual	24	1.041	0.043		
Total	35	52.557			

Figure 10C

	df	Sum Sq	Mean Sq	F-value	P-value
Treatment	7	49444.367	7064.195	925.741	< 0.0001
Residual	32	244.187	7.631		
Total	39	49693.554			

Supplemental Table S2. ANOVA tables

Sum Sq = Sum of squares; df = degrees of freedom; Mean Sq = Mean Squares

Figure 10D

	df	Sum Sq	Mean Sq	F-value	P-value
Treatment	7	63250.236	9035.748	4545.991	< 0.0001
Residual	32	63.604	1.988		
Total	39	63313.840			

Supplemental Figure 2C

	df	Sum Sq	Mean Sq	F-value	P-value
Treatment	14	33714.311	2408.165	238.695	< 0.0001
Residual	30	302.667	10.089		
Total	44	34016.978			

Supplemental Figure 2D

	df	Sum Sq	Mean Sq	F-value	P-value
Treatment	14	55972.578	3998.041	368.672	< 0.0001
Residual	30	325.333	10.844		
Total	44	56297.911			

Supplemental Figure 3A

	df	Sum Sq	Mean Sq	F-value	P-value
Treatment	11	20892.750	1899.341	230.223	< 0.0001
Residual	24	198.000	8.250		
Total	35	21090.750			

Supplemental Table S2. ANOVA tables

Sum Sq = Sum of squares; df = degrees of freedom; Mean Sq = Mean Squares

Supplemental Figure 3B

	df	Sum Sq	Mean Sq	F-value	P-value
Treatment	11	25614.972	2328.634	188.634	< 0.0001
Residual	24	296.667	12.361		
Total	35	25911.639			

Supplemental Figure 4

	df	Sum Sq	Mean Sq	F-value	P-value
Treatment	11	364557.190	33141.563	166.696	< 0.0001
Residual	24	4771.550	198.815		
Total	35	369328.740			

Supplemental Figure 5A

	df	Sum Sq	Mean Sq	F-value	P-value
Treatment	8	37219.333	4652.417	694.007	< 0.0001
Residual	18	120.667	6.704		
Total	26	37340.000			

Supplemental Figure 5B

	df	Sum Sq	Mean Sq	F-value	P-value
Treatment	8	42718.000	5339.750	466.580	< 0.0001
Residual	18	206.000	11.444		
Total	26	42924.000			
